# Real-Time, Minimally Invasive Oxygen Sensing via Luminescence Loss in Singlet Oxygen-Resistant Triplet–Triplet Annihilation Upconversion Nanocapsules

**DOI:** 10.1021/acsnano.6c09585

**Published:** 2026-08-13

**Authors:** Maria Micheva, Yuri Avlasevich, Kerstin Steinbrink, Stanislav Baluschev, Katharina Landfester

**Affiliations:** † Department of Physical Chemistry of Polymers, Max Planck Institute for Polymer Research, Ackermannweg 10, Mainz 55128, Germany; ‡ Department of Medical Chemistry and Biochemistry, Faculty of Medicine, Medical University—Sofia, 1 Saint Georgi Sofiyski Street, Sofia 1431, Bulgaria; § Department of Dermatology, University Hospital Münster, University of Münster, Von-Esmarch-Str. 58, Münster 48149, Germany; ∥ Optics and Spectroscopy Department, Faculty of Physics, Sofia University “Saint Kliment Ohridski”, 5 James Bourchier Blvd., Sofia 1164, Bulgaria

**Keywords:** singlet oxygen, upconversion, normoxia, hypoxia, nanocapsules

## Abstract

Oxygen concentration in the extracellular microenvironment plays a critical role in regulating metabolism and disease progression, particularly under hypoxic conditions. Here, we introduce a radically new optical sensing strategy for real-time, minimally invasive monitoring of dissolved oxygen in the extracellular aqueous microenvironment. Instead of relying on the oxygen-dependent quenching of phosphorescence or delayed fluorescence lifetimes, which is commonly used in existing sensors, we demonstrate that the local cumulative loss of upconverted fluorescence intensity in triplet–triplet annihilation upconversion (TTA-UC) nanocapsules provides a highly sensitive, one-to-one correspondence with the local oxygen concentration. This method enables an oxygen sensing dynamic range from normoxia (160 mmHg) to deep hypoxia (1.5 mmHg), achieving a detection limit of 45 nM. The sensing platform combines nanoconfined TTA-UC chromophores with sacrificial singlet oxygen scavengers (SSOS) within a hydrophobic nanocapsule core, ensuring complete chemical sequestration of photogenerated singlet oxygen. Furthermore, the use of a hydrophobic surfactant *n*-octadecyl trimethylammonium chloride (OTAC) minimizes free surfactant in the aqueous phase, drastically reducing cytotoxicity. With excitation intensities as low as 6.6 W cm^–2^ (λ = 631 nm), radiation stress is kept negligible. Crucially, the method requires no signal averaging, enabling real-time, high-fidelity oxygen monitoring without artifacts from dynamic equilibria. This approach offers a transformative tool for studying oxygen dynamics in living cells and tissues.

## Graphical abstract

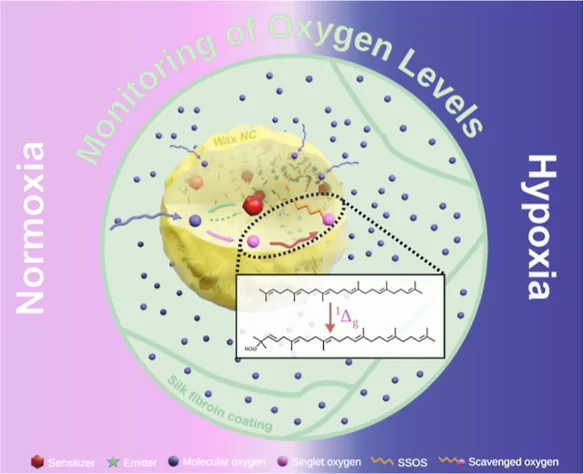

## Introduction

Oxygen is a fundamental regulator of cellular function, influencing metabolism, signaling pathways, and disease mechanisms—where hypoxia profoundly alters immune mechanisms, generally driving immunosuppression within tumor microenvironments and increasing susceptibility to infection.^1^ So, tumor hypoxia occurs when rapidly growing tumors outpace their blood supply,^2^ leading to low oxygen levels that stabilize hypoxia-inducible factor 1-alpha (HIF-1α) and activate genes promoting angiogenesis, metabolic reprogramming, and survival. In the context of infections, hypoxia modulates the activity of viruses, like Hepatitis C and Dengue, via upregulation of anaerobic glycolysis and stabilization of hypoxia-inducible factors (HIF-1α/2α).^3–6^

Real-time, spatially resolved monitoring of oxygen in the extracellular aqueous microenvironment is essential for understanding both physiological and pathological processes. Optical sensing methods are particularly advantageous due to their noninvasiveness, compatibility with live-cell imaging, and ability to provide two-dimensional (2D) spatial maps of oxygen distribution.^7–9^ Among these, phosphorescence quenching by molecular oxygen has become a gold standard, enabling lifetime-based quantification across tissues.^10,11^ Complementary approaches use oxygen-sensitive dyes immobilized in hydrogels to report average oxygen consumption rates in cell cultures.^12,13^

However, these methods face key limitations. Phosphorescence lifetime measurements often require extensive signal averaging, which can mask transient dynamics and introduce artifacts. Moreover, many optical sensing schemes—especially those based on triplet-state quenching—inevitably generate singlet oxygen (^1^O_2_) during excitation. This reactive species causes oxidative damage to both the sensor and surrounding biological systems, leading to photobleaching, irreversible sensor degradation, and unintended biological effects.^14–18^

Triplet–triplet annihilation upconversion (TTA-UC) offers a promising alternative. It operates under low excitation intensities (typically ∼1 W × cm^–2^) and uses red light (λ > 630 nm), thereby minimizing photodamage. TTA-UC relies on a sensitizer–emitter pair: the sensitizer absorbs light and transfers triplet energy to the emitter, which then undergoes triplet–triplet annihilation to produce delayed fluorescence at higher energy.^19,20^ While this process is inherently sensitive to oxygen, the dependence of delayed fluorescence lifetime on oxygen concentration is not stable over time due to the dynamic interplay between oxygen diffusion, singlet oxygen generation, and its chemical scavenging.^21^

Here, we present a fundamentally new sensing paradigm: instead of measuring lifetime changes, we monitor the cumulative loss of the upconverted fluorescence intensity over time. This parameter is highly sensitive to local oxygen levels and remains stable once a dynamic equilibrium is reached. We further engineer the nanocapsule architecture to prevent singlet oxygen diffusion into the aqueous environment and eliminate cytotoxic surfactants, enabling truly minimally invasive long-term oxygen monitoring.

## Results and Discussion

### Design of a Singlet Oxygen-Immune TTA-UC Nanosensor

We developed a nanocapsule-based TTA-UC system (diameter: 120 nm, see Figure S1) encapsulating a palladium­(II) porphyrin sensitizer (PdTBP, synthesis see Figure S3) and a perylene emitter (BDMP) in a hydrophobic core composed of beeswax (40 wt %), squalene (40 wt %), and rice bran oil (20 wt %). On the one hand, the solubility of ground-state oxygen in the oil phase (squalene and rice bran oil) of the NC core is more than 10 times higher than the solubility of oxygen in the surrounding aqueous phase.^22^ On the other hand, beeswax exhibits exceptionally high barrier properties toward ground-state oxygen and water vapor.^23^ Proper balance between the barrier properties (wax content) and scavenging ability (oil–content) is required.^24^

Crucially, it also hosts sacrificial singlet oxygen scavengers (SSOS)—squalene and rice bran oil—which chemically bind ^1^O_2_ generated during excitation, preventing its diffusion into the surrounding aqueous phase.

To stabilize the oil-in-water nanocapsules, we replaced conventional water-soluble surfactants (e.g., Triton X-100 (2-[4-(2,4,4-trimethylpentan-2-yl)­phenoxy]­ethanol), SDS (sodium dodecyl sulfate)) with *n*-octadecyl trimethylammonium chloride (OTAC), a hydrophobic surfactant with low water solubility (1.759 mg/L at 25 °C or less than 0.02% wt, purity = 95%). This ensures minimal free surfactant in the extracellular environment, drastically reducing cytotoxicity while maintaining colloidal stability (Figure S2). Figure 1 illustrates the energy-level diagram of the TTA-UC process in both oxygen-free (hypoxic) and oxygen-saturated (normoxic) environments (for more information, see also Figure S9). The inset shows the molecular structures of the PdTBP sensitizer and BDMP emitter. The process involves triplet energy transfer (TTT), residual phosphorescence (rPh), and singlet oxygen generation (ET), with delayed fluorescence (dF) arising from triplet–triplet annihilation (TTA). This diagram is essential for understanding the origin of the signals used in our sensing strategy. The home-built experimental setup is shown in Supporting Information (Figure S6).

**Figure 1:**
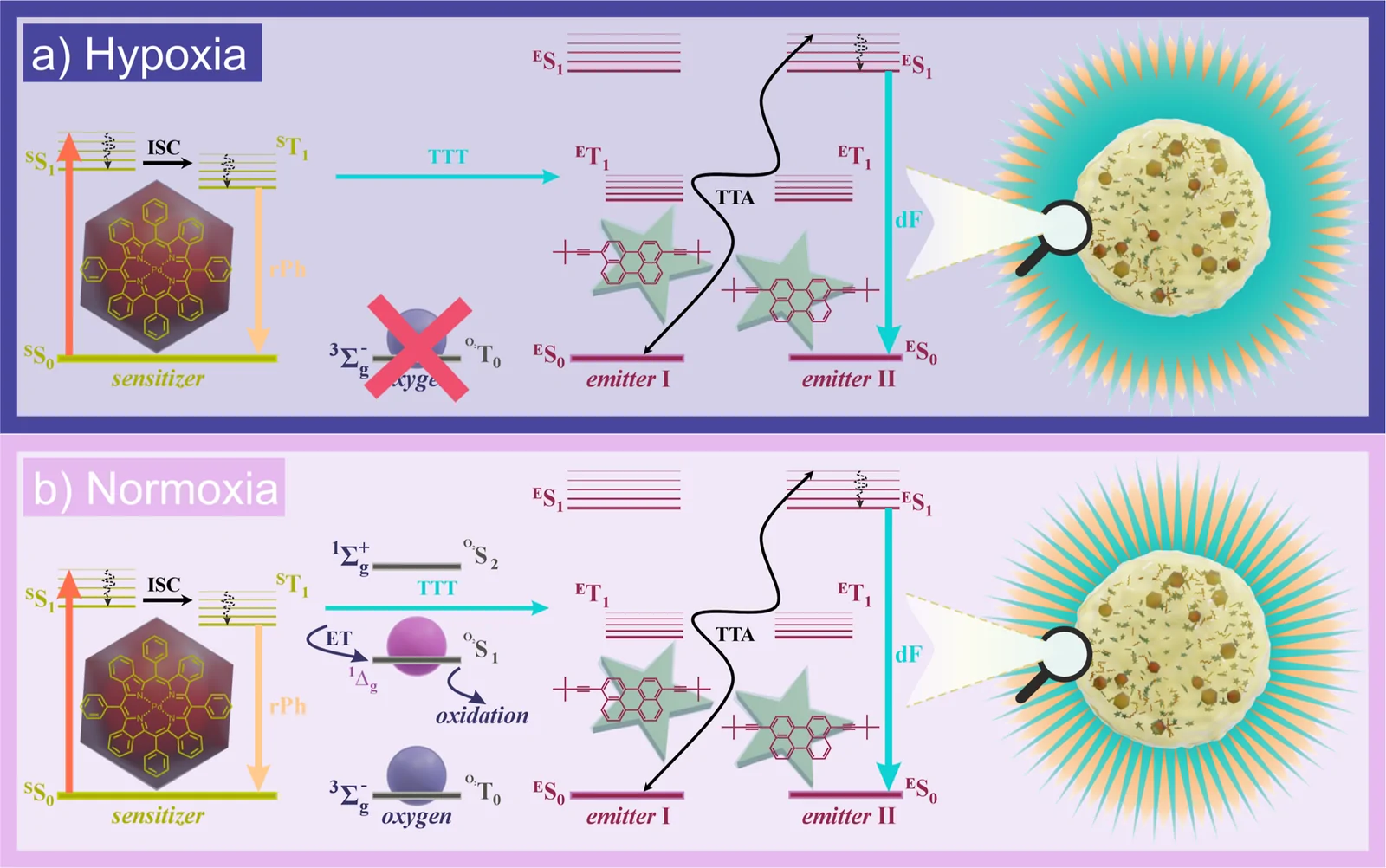
Schematic energy-level diagram of the TTA-UC process in (a) an oxygen-free (hypoxic) and (b) an oxygen-saturated (normoxic) environment. Insets: structure of meso-tetraphenyl-tetrabenzo­[2,3]­porphyrin palladium­(II) (PdTBP) sensitizer (left), structure of (3,9(10)-bis­(3,3-dimethylbutyn-1-yl)­perylene) (BDMP) emitter (right). The process involves triplet energy transfer (TTT), residual phosphorescence (rPh), and singlet oxygen generation (ET), with delayed fluorescence (dF) arising from triplet–triplet annihilation.

### Dynamic Behavior of Delayed Fluorescence: A New Sensing Metric

The time-resolved intensity of delayed upconverted fluorescence (dF, blue arrow) and sensitizer residual phosphorescence (rPh, orange arrow) in an oxygen-saturated aqueous environment is shown in Figure S4 (Supporting Information). A magnified view of the first 25 excitation pulses (Figure S5) reveals a key kinetic insight: (1) the residual phosphorescence (rPh) is intramolecular and persists even after the first few pulses (nonzero intensity at pulse 4) and (2) the delayed fluorescence (dF) is intermolecular and is initially suppressed due to oxygen quenching (complete suppression during the first 3 pulses). This demonstrates that, in an oxygen-saturated soft matter matrix, the diffusion-controlled TTA-UC process is outpaced by the generation of singlet oxygen and the emission of rPh. Crucially, dF is much more sensitive to ground-state oxygen than rPh, making it an ideal reporter for real-time oxygen sensing (Figures S7 and S8).

Figure 2 presents the time-resolved delayed fluorescence (dF) dynamics under extreme oxygen conditions. In Figure 2a (hypoxia) and b (normoxia), a schematic of the experimental setup is shown with TTA-UC nanocapsules dispersed in a supporting silk fibroin hydrogel, excited with pulsed red light (631 nm). In Figure 2c, the dF lifetime is given as a function of time under normoxia (160 mmHg, pink circles) and glovebox (4 ppm of O_2_, purple circles), showing a monotonic increase from ∼20 to 80 μs under normoxic conditions due to oxygen consumption in the nanocapsule core. The time-resolved dF intensity at early (pulse N25, 150 ms after start) and late (pulse N520, 3.12 s after start) times is shown in Figure 2d,e, respectively. These data illustrate the progressive recovery of the dF signal in normoxia as dynamic equilibrium is approached. In an oxygen-free environment, the dF lifetime stabilizes at 80 ± 2.5 μs (Figure 2c, purple circles), consistent with the intrinsic decay of the excited emitter state. In contrast, under normoxic conditions, the dF lifetime increases monotonically from ∼20 μs at the start of excitation to 80 μs after ∼2.58 s (Figure 2c, pink circles). This behavior arises because each excitation cycle consumes oxygen within the nanocapsule core, reducing local quenching. Once a dynamic equilibrium is reached where oxygen influx balances consumption, the system behaves as if oxygen-free.

**Figure 2:**
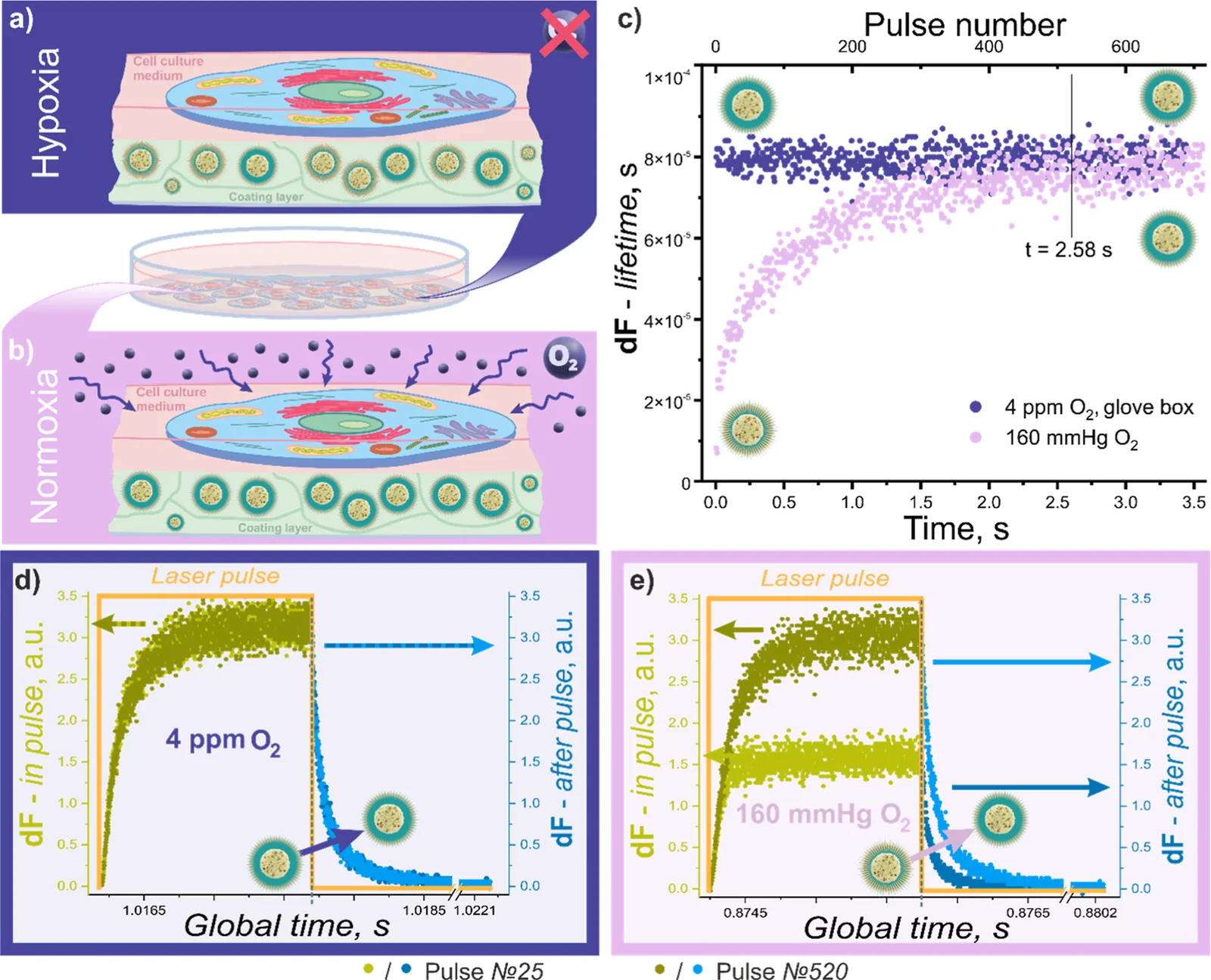
Potential application example for time-resolved delayed fluorescence (dF) dynamics under extreme oxygen conditions. (a,b) Schematic of the experimental setup: TTA-UC nanocapsules dispersed in supporting silk fibroin hydrogel, excited with pulsed red light (631 nm). (c) dF lifetime as a function of time under normoxia (160 mmHg, pink circles) and in a glovebox (4 ppm of O_2_, purple circles). The lifetime increases under normoxia due to oxygen consumption in the nanocapsule core. (d,e) Time-resolved dF intensity at two time points and two oxygen concentrations: (d) early pulse (N25, 150 ms after start, light green points) is compared with the late pulse (N520, 3.12 s after start, dark green points), hypoxic environment; and (e) the signal suppression in normoxia is pronounced at early times but diminishes over time.

This time-dependent lifetime shift renders lifetime-based sensing unreliable: averaging over multiple pulses would yield misleading results as the system is not in equilibrium at the start. Instead, we propose a new metric: the integrated delayed fluorescence during excitation, $I_{in}^{dF}\left(n\right)$ , defined as the area under the dF signal during the *n*-th excitation pulse (Figure 3, green area).

**Figure 3:**
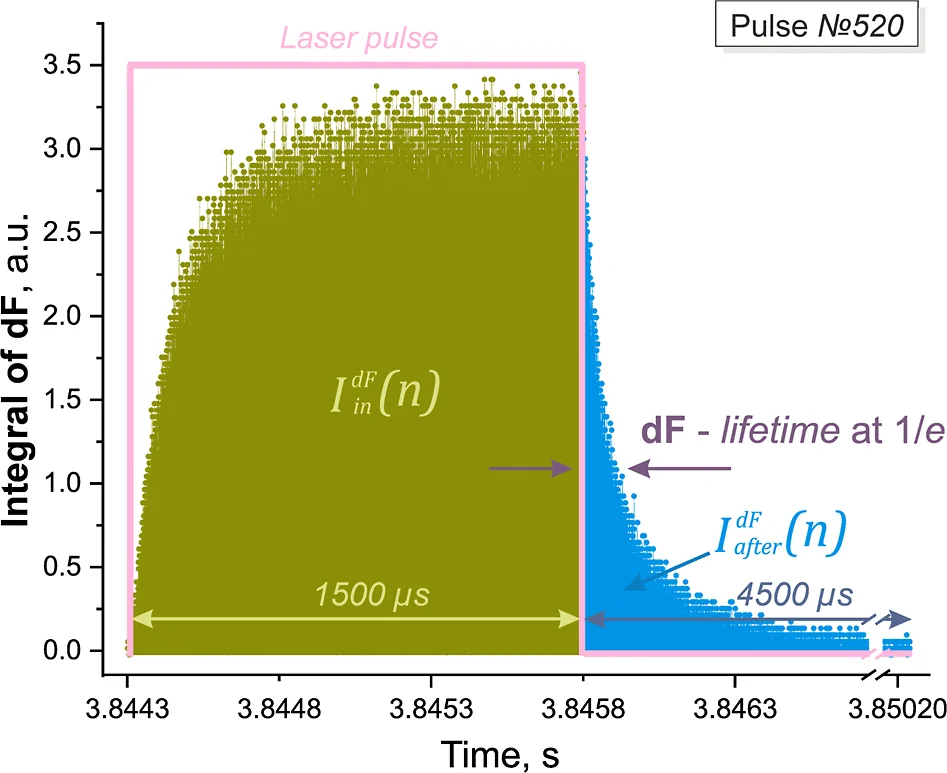
Definition of the integrated delayed fluorescence signals: Schematic of the excitation pulse (duration of τ_pulse_ = 1.5 ms) and the observation window; the green area represents the integral of the dF signal during the excitation pulse, $I_{in}^{dF}\left(n\right)$ . The blue area represents the integral after the pulse, $I_{after}^{dF}\left(n\right)$ . The former is used as the primary sensing parameter due to its stable, reproducible behavior.

Figure 3 defines the integrated delayed fluorescence signals: schematic of the excitation pulse (duration τ_pulse_ = 1.5 ms) and observation window. The green area represents the integral during the pulse $I_{in}^{dF}\left(n\right)$ . The blue area represents $I_{after}^{dF}\left(n\right)$ , the integral after the pulse. The in-pulse integral is used as the primary sensing parameter due to its stable, reproducible behavior.

This approach enables real-time, single-shot oxygen monitoring without artifacts from dynamic equilibria, which is a transformative step forward for optical sensing.

### Quantitative Oxygen Sensing via Global Luminescence Loss

We define the loss of nanocapsule luminescence as

$$L_{pO_{2}}^{dF}=\sum_{n=1}^{N_{ss}}\left[I_{ss}^{dF}\left(N_{ss}\right)-I_{in}^{dF}\left(n,pO_{2}\right)\right]$$

where $I_{in}^{dF}\left(n,pO_{2}\right)$ is the in-pulse integral at the n-th pulse, and $I_{ss}^{dF}\left(N_{ss}\right)$ is the steady-state value, for a given oxygen partial pressure pO_2_.

Figure 4 shows the time-dependent behavior of integrated delayed fluorescence signals: (a) time-resolved dF intensity at different pulses (Figure 4a), $I_{in}^{dF}\left(n\right)$ reaches a plateau after ∼2.58 s (Figure 4b), and $I_{after}^{dF}\left(n\right)$ does not stabilize (Figure 4c). The in-pulse integral is therefore the most reliable metric for oxygen sensing.

**Figure 4:**
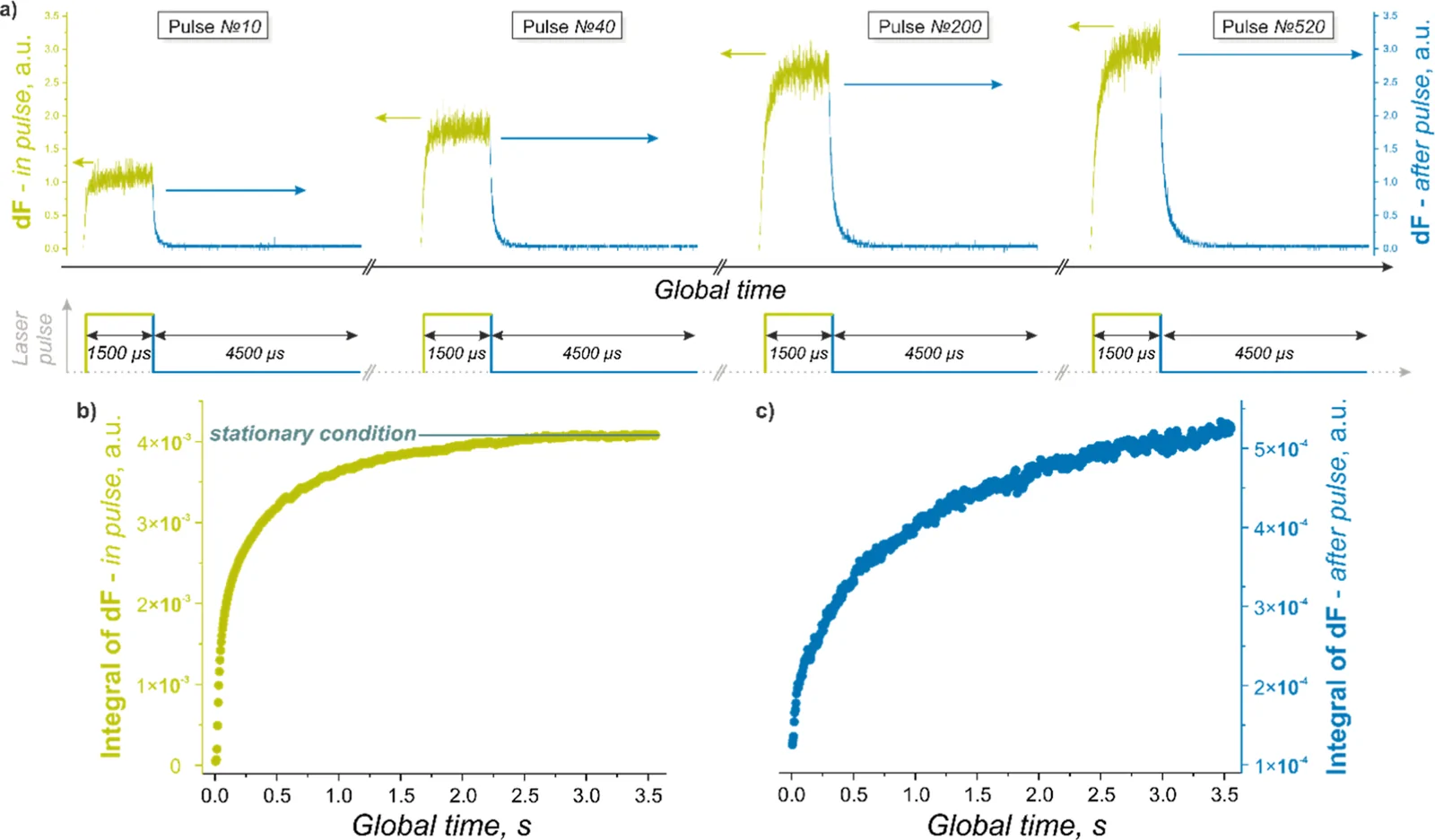
Time-dependent behavior of integrated delayed fluorescence signals: (a) time-resolved dF intensity at different excitation pulses: pulse 10 (54 ms), 40 (234 ms), 200 (1.194 s), and 520 (3.12 s). (b) $I_{in}^{dF}\left(n\right)$ , (light green circles) reaches a plateau after ∼2.58 s. (c) $I_{after}^{dF}\left(n\right)$ , (dark blue circles) does not stabilize. The in-pulse integral is therefore the most reliable metric for oxygen sensing.

Figure 5 demonstrates the critical influence of excitation pulse duration on sensor stability. The rate of oxygen diffusion is governed by the partial pressure difference between the nanocapsules and the surrounding water microenvironment.^21^ At the onset of optical excitation (i.e., *t* = 0), the partial pressure difference between the nanocapsule core and the surrounding water is maximal, resulting in the highest oxygen influx. Once steady state is reached (i.e., *t* = τ_ss_), the ground-state oxygen flux from the microenvironment stabilizes at a constant value, reflecting the balance between influx and consumption. The optimal excitation intensity (at constant pulse duration and constant repetition rate) is shown in Supporting Information (Figure S10).

**Figure 5:**
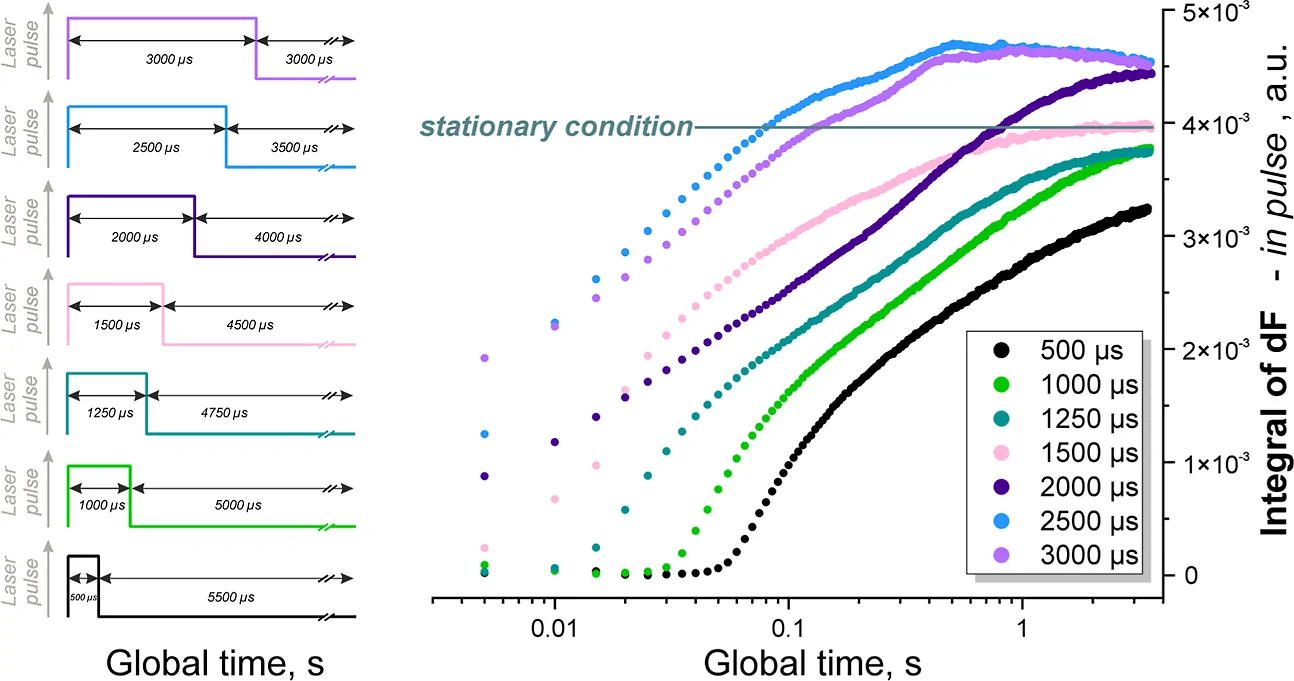
Dependence of the integrated dF signal on excitation pulse duration. Temporal evolution of $I_{in}^{dF}\left(n\right)$ for different pulse durations (τ_pulse_): 0.5 ms (black), 1 ms (dark green), 1.25 ms (light green), 1.5 ms (pink), 2 ms (dark violet), 2.5 ms (light blue), and 3 ms (violet). All measurements use the same pulse period (τ_pp_ = 6 ms). Longer pulses lead to rapid ^1^O_2_ accumulation and sensor degradation (evident in the drop of $I_{in}^{dF}\left(n\right)$ ), while shorter pulses allow oxygen influx and stable operation.

To ensure consistent experimental conditions across all measurements, the excitation pulse periods were kept identical. The chemical binding of photogenerated singlet oxygen is also diffusion-controlled. If the local concentration of ^1^O_2_ becomes too high, both the SSOS materials and the TTA-UC chromophores may undergo nonselective oxidation. For τ_pulse_ = 2 ms (dark violet), 2.5 ms (light blue), and 3 ms (violet), the $I_{in}^{dF}\left(n\right)$ signal increases initially, but then decreases sharply, indicating irreversible degradation of the TTA-UC system due to nonselective oxidation. The significant decrease of the $I_{in}^{dF}\left(n\right)$ values demonstrates the loss of optically active materials (sensitizer and/or emitter molecules) as a result of photo-induced oxidation.^17,18^

In contrast, when the excitation pulse duration is too short (e.g., τ_pulse_ = 0.5, 1, or 1.25 ms), the rate of oxygen consumption is below the rate of oxygen influx and initial oxygen concentration. As a result, the system cannot reach a steady state, and the $I_{in}^{dF}\left(n\right)$ signal remains unstable.

Steady-state conditions are achieved when the rate of oxygen influx balances the rates of consumption and scavenging.

This is illustrated in Figure 2c (τ_pulse_ = 1.5 ms, pink circles) and Figure 5 (pink circles), where $I_{in}^{dF}\left(n\right)$ reaches a stable plateau after τ_ss_ ∼2.58 s. It is important to note that the time required to reach steady state (τ_ss_) is drastically dependent on the oxygen concentration in the surrounding water microenvironment.

Figure 6 provides the definitive proof of our new sensing paradigm. In Figure 6a, the time-dependent evolution of $I_{in}^{dF}\left(n\right)$ is shown across a logarithmic range of oxygen partial pressures—from normoxia (160 mmHg) to deep hypoxia (1.5 mmHg). Despite the complex transient dynamics, the system reaches a stable plateau at each oxygen level, confirming the robustness of the in-pulse integral as a sensing parameter. Crucially, Figure 6b presents the calibration curve: the global luminescence loss $L_{{pO}_{2}}^{dF}$ exhibits a one-to-one correspondence with the local oxygen concentration. This calibration spans a 100-fold dynamic range and achieves a detection limit of 45 nM, far surpassing the sensitivity of lifetime-based methods. Figure 6c offers a visual representation of the global luminescence loss across oxygen regimes: from glovebox (4 ppm of O_2_, purple) to normoxia (160 mmHg, violet). The filled areas illustrate the magnitude of signal loss, which increases by over 14-fold between deep hypoxia and normoxia, demonstrating the superior sensitivity of our approach. Together, Figure 6 establishes that the cumulative loss of upconverted fluorescence luminescence is not just a measurable signal, but a quantifiable, reliable, and highly sensitive metric for real-time oxygen sensing. This minimally invasive approach for monitoring oxygen dynamics in living cells enables the investigation of ROS production from intracellular prodrugs.^25^

**Figure 6:**
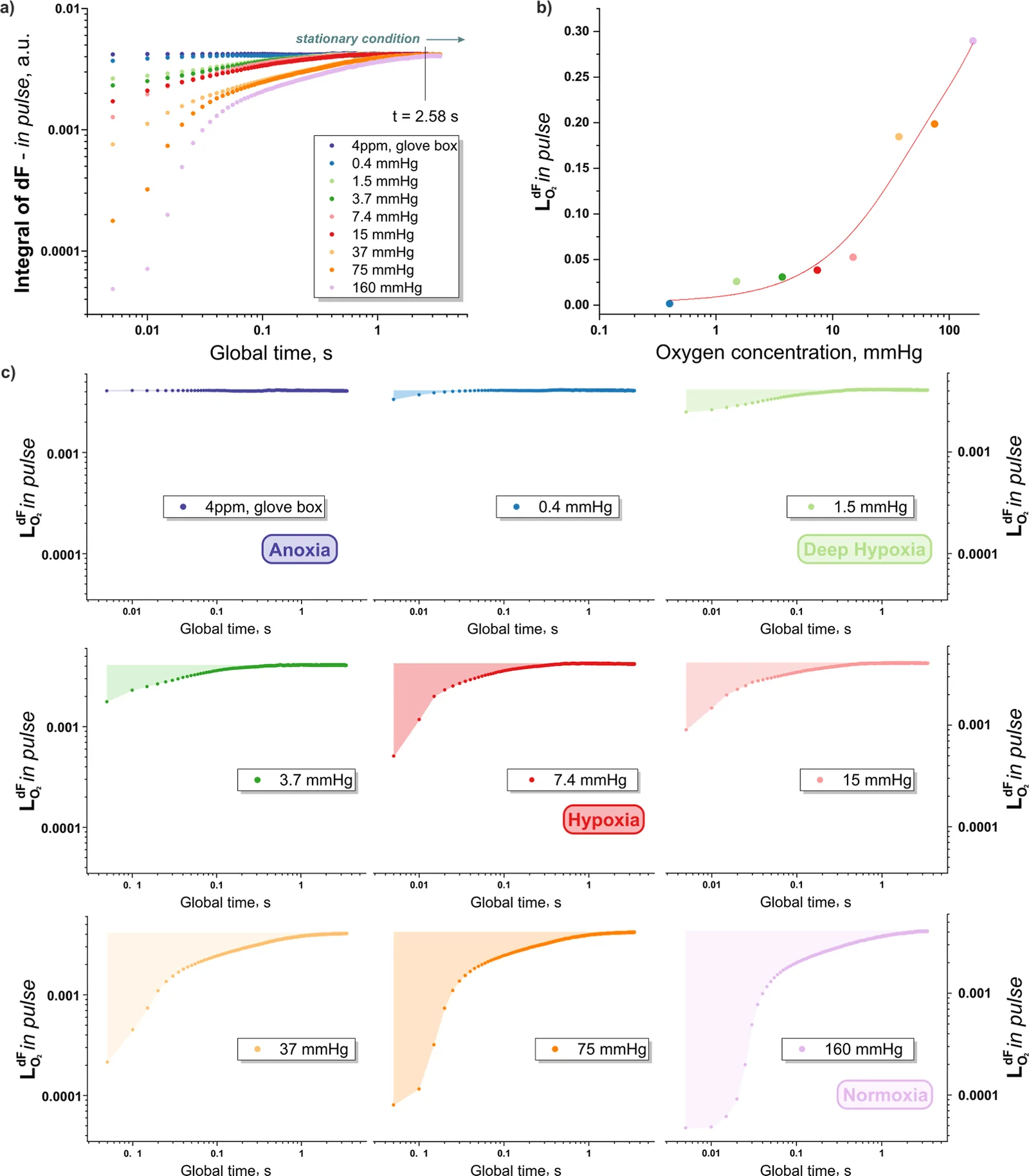
High-sensitivity, wide-dynamic-range oxygen sensing via luminescence loss. (a) Time-dependent $I_{in}^{dF}\left(n\right)$ under varying oxygen partial pressures (logarithmic scale: 160 → 1.5 mmHg). The time to reach steady state (τ_ss_) depends on oxygen concentration. (b) Calibration curve: the luminescence loss $L_{{pO}_{2}}^{dF}$ as a function of local oxygen concentration. The experimental data can be fitted with a double-exponential function (red curve in b), as shown in Figure S16 of the Supporting Information. (c) Visual representation of the global luminescence loss across oxygen regimes: glovebox (4 ppm of O_2_, purple), deep hypoxia (1.5 mmHg, light green), hypoxia (7.4 mmHg, red), and normoxia (160 mmHg, violet). The filled areas indicate the extent of signal loss.

The global luminescence loss, $L_{{pO}_{2}}^{dF}$ reported in this study exhibits a one-to-one correlation with the local oxygen concentration in the so-called nonequilibrium regime, i.e., before the dF emission reaches a steady-state value under constant optical excitation. Furthermore, the TTA-UC process is diffusion-controlled, with its kinetics determined by the diffusion of molecular oxygen within the NC core, the diffusion of molecular oxygen across the NC shell, and the scavenging rate of the generated singlet oxygen through photooxidation of the SSOS material.

The delayed fluorescence (dF), resulting from the process of nanoconfined TTA-UC in an oxygen-rich environment, does not obey the Stern–Volmer equation directly because oxygen quenches the sensitizer triplet population, not the emitted fluorescence itself. As shown in Figure S11, even after reaching a steady-state dF emission under constant optical excitation, the intensity dependence (of the nanoconfined TTA-UC system) is superlinear: the experimental data are well approximated with a power law $I_{TTA‐UC}=a\times I_{exc}^{b}$ , where the parameter **b** = 1.11. This superlinear intensity dependence is demonstrated for many energetically optimized TTA-UC molecular couples emitter/sensitizer.^26^

Among others, the global luminescence loss $L_{{pO}_{2}}^{dF}$ of the TTA-UC process (in the aqueous microenvironment) depends on a multitude of environmental parameters, including (i) the composition of the cell culture medium; (ii) the degree to which physiological conditions—such as pH, osmolarity, and ion concentrations—are replicated for cell ensembles; (iii) the sample temperature; (iv) variations in the nanocarrier (NC) solid content; (v) the excitation intensity; and (vi) sequential oxygen measurements performed over a defined time interval. All of these environmental parameters influence the TTA-UC efficiency either directly, by affecting the photophysical properties of the system (e.g., nanocarrier solid content, temperature, and excitation intensity), or indirectly, by modulating the solubility and diffusion of molecular oxygen in the aqueous microenvironment (e.g., through changes in the solvent ionic strength). To describe this complex multiparametric behavior and demonstrate the robustness and predictable tunability of the proposed oxygen calibration curves, we adopt a Stern–Volmer-like metric based on the ratio of the global luminescence loss, $L_{{pO}_{2}}^{dF}$ , at a given oxygen concentration to the global delayed fluorescence intensity, g*I* ^dF^, measured at steady state in the oxygen-free NC core. The global delayed fluorescence g*I* ^dF^ is defined by $gI^{dF}=I_{ss}^{dF}\left(N_{ss}\right)\times N_{ss}$ , where *N*_ss_ = τ_ss_/τ_pp_ represents the number of successive excitation pulses necessary to reach steady-state dF emission.

The sensing stability and resistance to biological interference of the reported sensing system are demonstrated in Figure S13a–c: partial replacement of the continuous water phase with Dulbecco’s phosphate buffered saline (PBS), Dulbecco’s modified eagle medium (DMEM), or pure water, while maintaining a constant NC solid content, does not significantly alter the TTA-UC response. A substantially different response is observed only when the total ethanol (EtOH) concentration is increased to levels sufficient to eliminate biological contamination (Figure S13d). Nevertheless, exposure to such EtOH concentrations will be limited to a short duration, followed by washing of the hydrogels incorporating the sensing NC.

As shown in Figure S14 f, for the physiologically important temperature region, from *T* = 32 °C up to *T* = 38 °C, the variation of the ratio of the global luminescence loss, $L_{{pO}_{2}}^{dF}$ (at a given oxygen concentration) to the global delayed fluorescence intensity g*I* ^dF^ is less than 5% (the growth follows a stable monotonic trend). This demonstrates the ability for controllable tuning of the $L_{{pO}_{2}}^{dF}$ —oxygen calibration curves.

Another important parameter is the ability to perform sequential oxygen sensing applying the same (or controllably tuned) calibration curve. As shown in Figure S12f, the $L_{{pO}_{2}}^{dF}$ —oxygen calibration curves can be tuned in a controlled and readily automatable manner, provided that the excitation sequence number is known. The total observation time can be optimized following two strategies—either by increasing the amount of SSOS—materials, or alternatively—by drastically reducing the oxygen permeability of the NC-shell by introducing natural waxes with higher melting temperature, such as carnauba wax—Figure S15e.

### Robustness and Practical Advantages

The true power of a sensing platform lies not only in its sensitivity but also in its real-world applicability. Our TTA-UC nanocapsule system excels not just in precision but also in a suite of practical features that make it uniquely suited for biological applications.

#### First Advantage: No Signal Averaging Required

Unlike conventional lifetime-based methods that demand extensive signal averaging—often over hundreds of pulses—our approach delivers a stable, high-signal output within seconds. The integrated delayed fluorescence signal is strong enough to enable single-shot or real-time measurements, making it ideal for capturing transient oxygen dynamics in living cells and tissues.

#### Second Advantage: Ultralow Excitation Intensity

With a maximum excitation intensity of only 6.6 W cm^–2^ (631 nm), our system operates well below the biological safety threshold (10 W cm^–2^ ≈100 suns). This minimal photo—stress enables long-term, continuous monitoring without compromising cell viability, which is critical for studying dynamic processes in living systems. Figure 5 further reveals that excitation pulse duration critically affects system stability. Long pulses (τ_pulse_ > 2 ms) lead to rapid ^1^O_2_ accumulation and subsequent sensor degradation (violet, light blue, and dark violet curves), while optimal duration pulses (τ_pulse_ = 1.5 ms) allow sufficient time for oxygen influx and scavenging, enabling stable steady-state operation (pink curves). This highlights the importance of balancing excitation and diffusion kinetics.

#### Third Advantage: Complete Singlet Oxygen Sequestration

By combining nanoconfined TTA-UC chromophores with sacrificial singlet oxygen scavengers (SSOSs), every photogenerated ^1^O_2_ is chemically trapped within the hydrophobic nanocapsule core. No ^1^O_2_ leaks into the surrounding aqueous environment, eliminating oxidative damage to both the sensor and biological samples, ensuring both sensor stability and biological fidelity.

#### Fourth Advantage: Exceptionally Low Cytotoxicity

The use of *n*-octadecyltrimethylammonium chloride (OTAC), a hydrophobic surfactant with extremely low water solubility (1.759 mg/L), ensures that virtually no free surfactant remains in the aqueous phase. This dramatically reduces cytotoxicity compared to conventional water-soluble surfactants (e.g., Triton X-100, SDS), making the system highly biocompatible as shown for NIH 3T3 fibroblast cells (see Figure S2).

#### Fifth Advantage: High Reproducibility across Batches

The response is consistent across different production batches, which is a crucial requirement for reliable, standardized research. This reproducibility ensures that the results are not only sensitive but also robust and trustworthy.

In summary, this platform is not just scientifically innovative but also practically viable, biocompatible, and ready for real-world biological applications. It enables real-time, long-term oxygen monitoring without compromising cell health, making it a transformative tool for studying oxygen-dependent processes in living cells and tissues.

## Conclusions

We present a transformative approach to oxygen sensing in aqueous microenvironments: monitoring the cumulative loss of TTA-UC luminescence rather than lifetime changes. This method achieves unprecedented sensitivity (45 nM), a vast dynamic range (1.5–160 mmHg), and eliminates the need for signal averaging. By combining nanoconfined TTA-UC chromophores with sacrificial singlet oxygen scavengers and a hydrophobic surfactant, we ensure complete sequestration of reactive oxygen species and minimal biological perturbation.

This platform enables real-time, long-term, and minimally invasive monitoring of oxygen dynamics in living cells and tissues—particularly valuable in hypoxic conditions relevant to cancer, infection, and ischemia. With its robustness, sensitivity, and biocompatibility, this technology opens new avenues for studying oxygen-dependent biological processes at the microscale.

## Methods/Experimental Section

### Nanocapsules Production

Toluene solutions of PdTBP (10^–4^ M, 1.5 mL) and π-extended perylene dye BDMP (10^–3^ M, 0.75 mL) were combined in a 10 mL flask, and the solvent was evaporated under vacuum to dryness. Squalene (200 mg), beeswax (200 mg), and rice bran oil (100 mg) were combined in a 40 mL vial, melted at 70 °C and homogenized using an IKA Vortex Genius 3. Combined dyes were dissolved in ethyl acetate (3 × 0.5 mL) and added to the wax phase at elevated temperature (70 °C) to form a homogeneous solution. Then, a hot solution of OTAC (10 mL, 4% wt, 70 °C) was added and homogenized using an IKA Vortex Genius 3. Afterward, the hot mixture was sonicated with Branson Digital Sonifier 450 at 70 °C, 70% amplitude, 2 min, pulse/pause ratio −10 s/10 s, then cooled to room temperature and transferred into a 50 mL round-bottom flask, with a known weight. Ethyl acetate was slowly evaporated at room temperature, lowering the pressure step-wise from 250 mbar to 60 mbar. The final weight of the suspension should be less than 10 g. The resulting green suspension was transferred into a cellulose dialysis membrane (MWCO 14 kDa, Sigma-Aldrich GmbH) and dialyzed (at 36 °C) in 3 L of deionized water for 72 h; the water was changed every 24 h.

### Nanocapsule Characterization

Hydrodynamic diameter and size distribution were measured via dynamic light scattering (DLS) using a Malvern Zetasizer Nano S90 (90° scattering mode, 20 °C); zeta potential was measured in 10^–3^ M KCl solution using the same instrument.

For hydrodynamic diameter and surface charge characterization of the samples, dynamic light scattering and zeta potential measurements were performed, and average data were obtained from at least three runs of the measurements. The zeta potential of the nanocarriers was measured in 10^–3^ M potassium chloride solution with a ZetaNanosizer S90 (Malvern Panalytical) at 20 °C. In all cases, similar zeta potentials with an average value of +52 mV (*n* ≥ 3) were determined. Dynamic light scattering (DLS) was used to define the average size and size distribution (∼120 nm, see Figure S1). Measurements were done on a Malvern Zetasizer Nano S90 (Malvern Panalytical) equipped with a detector at 90° scattering mode at 20 °C. The high positive zeta potential confirms colloidal stability and minimal aggregation.

### Confocal Laser Scanning Microscopy

Instrument: Leica TCS SP5 II system (Leica, Germany); Objective: HCX PL APO 40.0× 0.85 DRY.

### Sample Preparation

The bottoms of Ibidi μ-Slide 18-well plates were coated with a mixture consisting of 95% vol. silk fibroin solution (5% wt) and 5% vol. TTA-UC nanocapsule dispersion (10% wt solid content) and left at 4 °C overnight. Wells were washed with 70% vol. ethanol solution and sterile PBS solution several times in order to sterilize the surface and remove the excess of coating mixture.

### Cell Culture

Fibroblast cells (NIH 3T3, 40 000 per well) were seeded in the already coated Ibidi wells and incubated overnight in the presence of DMEM containing 10% FBS, 1% penicillin/streptomycin in 5% CO_2_ at 37 °C. The acquired images (Figure S2) reveal distinct z-positions for the sensing nanocapsules (NCs) and the fibroblast cells, confirming that the NCs remain embedded within the silk fibroin hydrogel. No cytotoxicity was observed after 24 h. The cells remained viable and adherent, confirming the biocompatibility of the OTAC-stabilized nanocapsules.

## Supplementary Material



## Notes

Nanocapsule characterization (DLS); confocal imaging of NIH 3T3 fibroblasts; synthesis of PdTBP; optical setup for time-resolved measurements; additional time-resolved delayed fluorescence and residual phosphorescence data; and calibration curves (PDF)

## References

[1] Suvac A., Ashton J., Bristow R. G. (2025). Tumour hypoxia in driving genomic
instability and tumour evolution. Nature Rev.
Canc..

[2] Low J. T., Kim Y., Matsushita M., Ho P.-C. (2025). Role of oxygen sensing and hypoxia-inducible
factors in orchestrating innate immune responses. Nature Immunol.

[3] Vassilaki N., Kalliampakou K. I., Kotta-Loizou I., Befani C., Liakos P., Simos G., Mentis A. F., Kalliaropoulos A., Doumba P. P., Smirlis D., Foka P., Bauhofer O., Poenisch M., Windisch M. P., Lee M. E., Koskinas J., Bartenschlager R., Mavromara P. (2013). Low oxygen
tension enhances hepatitis
C virus replication. J. Virol..

[4] Frakolaki E., Kaimou P., Moraiti M., Kalliampakou K. I., Karampetsou K., Dotsika E., Liakos P., Vassilacopoulou D., Mavromara P., Bartenschlager R., Vassilaki N. (2018). The Role of
Tissue Oxygen Tension in Dengue Virus Replication. Cells.

[5] Serebrovska Z. O., Chong E. Y., Serebrovska T. V., Tumanovska L. V., Xi L. (2020). Hypoxia, HIF-1α, and COVID-19:
from pathogenic factors to potential
therapeutic targets. Acta Pharmacol. Sin..

[6] Devaux C. A., Lagier J.-C. (2023). Unraveling the Underlying
Molecular Mechanism of ‘Silent
Hypoxia’ in COVID-19 Patients Suggests a Central Role for Angiotensin
II Modulation of the AT1R-Hypoxia-Inducible Factor Signaling Pathway. J. Clin. Med..

[7] Ogilby P. R. (2010). Singlet
oxygen: there is indeed something new under the sun. Chem. Soc. Rev..

[8] Wang X., Wolfbeis O. S., Meier R. J. (2013). Luminescent
probes and sensors for
temperature. Chem. Soc. Rev..

[9] Papkovsky D. B., Dmitriev R. I. (2013). Biological detection by optical oxygen sensing. Chem. Soc. Rev..

[10] Roussakis E., Li Z., Nichols A. J., Evans C. L. (2015). Oxygen-Sensing Methods in Biomedicine
from the Macroscale to the Microscale. Angew.
Chem., Int. Ed..

[11] Quaranta M., Borisov S. M., Klimant I. (2012). Indicators
for optical oxygen sensors. Bioanal. Rev..

[12] Agilent Seahorse XF Cell Mito Stress Test Report Generator (RRID:SCR_026230), http://www.agilent.com/en/product/cell-analysis/real-time-cell-metabolic-analysis/xf-analyzers (accessed May 19, 2026).

[13] Oroboros NextGen-O2k, https://www.oroboros.at/product/nextgen-o2k-all-in-one/, (accessed May 19, 2026).

[14] Nazarova N., Avlasevich Y., Landfester K., Baluschev S. (2019). All-Optical
Temperature Sensing in Organogel Matrices via Annihilation Upconversion. ChemPhotoChem.

[15] Shimizu N., Ito J., Kato S., Otoki Y., Goto M., Eitsuka T., Miyazawa T., Nakagawa K. (2018). Oxidation of squalene by singlet
oxygen and free radicals results in different compositions of squalene
monohydroperoxide isomers. Sci. Rep..

[16] Marsico F., Turshatov A., Peköz R., Avlasevich Y., Wagner M., Weber K., Donadio D., Landfester K., Baluschev S., Wurm F. R. (2014). Hyperbranched Unsaturated
Polyphosphates
as Protective Matrix for Long-Term Photon Upconversion in Air. J. Am. Chem. Soc..

[17] Mehes G., Nomura H., Zhang Q., Nakagawa T., Adachi C. (2012). Enhanced electroluminescence
efficiency in a spiro-acridine derivative through thermally activated
delayed fluorescence. Angew. Chem., Int. Ed..

[18] Reis A., Spickett C. M. (2012). Chemistry of phospholipid oxidation. Biochim. Biophys. Acta Gen. Subj..

[19] Bharmoria P., Bildirir H., Moth-Poulsen K. (2020). Triplet–triplet
annihilation
based near infrared to visible molecular photon upconversion. Chem. Soc. Rev..

[20] Baluschev S., Miteva T., Yakutkin V., Nelles G., Yasuda A., Wegner G. (2006). Up-Conversion
Fluorescence: Non-coherent Excitation
by Sunlight. Phys. Rev. Lett..

[21] Byron Bird, R. ; Stewart, W. E. ; Lightfoot, E. N. Transport Phenomena, 2nd ed.; John Wiley & Sons, Inc., 2001; pp 567–572.

[22] Li Y., Chen F., Gao Z., Xiang W., Wu Y., Hu B., Ni X., Nishinari K., Fang Y. (2023). Influence of interfacial
properties/structure on oxygen diffusion in oil-in-water emulsions. Food Res. Internat..

[23] Donhowe G., Fennema O. (1993). Water Vapor and Oxygen
Permeability of Wax Films. JAOCS.

[24] Yang L., Paulson A. T. (2000). Effects of lipids
on mechanical and moisture barrier
properties of edible gellan film. Food Res.
Internat..

[25] Tang J. J., Liu Y., Xue Y., Jiang Z. Z., Chen B., Liu J. (2024). Endoperoxide-enhanced
self-assembled ROS producer as intracellular prodrugs for tumor chemotherapy
and chemodynamic therapy. Exploration.

[26] Filatov M. A., Baluschev S., Landfester K. (2016). Protection of densely populated excited
triplet state ensembles against deactivation by molecular oxygen. Chem. Soc. Rev..

