# Supplementary material for: Real-Time, Minimally Invasive Oxygen Sensing via Luminescence Loss in Singlet Oxygen-Resistant Triplet–Triplet Annihilation Upconversion Nanocapsules
Source: ACS Nano. 2026 Aug 13;20(33):23477–85. doi: 10.1021/acsnano.6c09585 (PMC13523760; doi:10.1021/acsnano.6c09585)
Supplement: Supplementary file 1 [file nn6c09585_si_001.pdf]

# Supporting Information

# Real-Time, Minimally Invasive Oxygen Sensing via Luminescence Loss in Singlet Oxygen-Resistant Triplet–Triplet Annihilation Upconversion Nanocapsules

Maria Micheva[†, ‡], Yuri Avlasevich[†], Kerstin Steinbrink[||], Stanislav Baluschev[†, §, *], Katharina Landfester[†*]

[†]Department of Physical Chemistry of Polymers, Max Planck Institute for Polymer Research, Ackermannweg 10, 55128 Mainz, Germany

[‡] Department of Medical Chemistry and Biochemistry, Faculty of Medicine, Medical University – Sofia, 1 Saint Georgi Sofiyski St., 1431 Sofia, Bulgaria

[||] Department of Dermatology, University Hospital Münster, University of Münster, Von-Esmarch-Str. 58, 48149 Münster, Germany

[§] Optics and Spectroscopy Department, Faculty of Physics, Sofia University "Saint Kliment Ohridski", 5 James Bourchier Blvd., 1164 Sofia, Bulgaria

**Table of Contents**

1. **Experimental Procedures**

Nanocapsule Characterization

Chemical Synthesis

Confocal Laser Scanning Microscopy (CLSM)

2. **Figures and Data**

**Figure S1:** DLS results of the average size and size distribution of the nanocapsules.

**Figure S2:** Confocal Imaging of NIH 3T3 Fibroblasts on Nanocapsule-Coated Surface.

**Figure S3:** Synthesis of PdTBP.

**Figure S4:** Time-Resolved dF and rPh Signals Under Normoxia

**Figure S5:** Magnified View of First 25 Excitation Pulses

**Figure S6:** Laser Setup for Time-Resolved Measurements

**Figure S7:** Time-Resolved rPh Signals (No Emitter Present)

**Figure S8:** Magnified rPh and dF Dynamics (First 25 Pulses)

**Figure S9:** dF Signal Evolution in Normoxia vs. Deep Anoxia

**Figure S10:** Dependence of $I_{in}^{dF}(n)$ signal on Excitation Intensity

**Figure S11:** Dependence of the dF on excitation intensity, after reaching the steady-state emission under constant optical excitation.

**Figure S12:** Dependence of the ratio $L_{pO_2}^{dF}$ to g$I^{dF}$ on the excitation sequence number.

**Figure S13:** Dependence of the ratio $L_{pO_2}^{dF}$ to g$I^{dF}$on the composition of the aqueous microenvironment

**Figure S14:** Dependence of the ratio $L_{pO_2}^{dF}$ to g$I^{dF}$ the temperature of the aqueous microenvironment.

**Figure S15:** Dependence of the $L_{pO_2}^{dF}$ on the excitation sequence number. Carnauba wax.

**Figure S16**: Double-exponential fit to the experimental data

**Figure S17**: Comparison of the excitation and emission spectrum of the process of TTA-UC and group of often used reporter fluorescent proteins.

## 3. Materials

## 1. Experimental Procedures

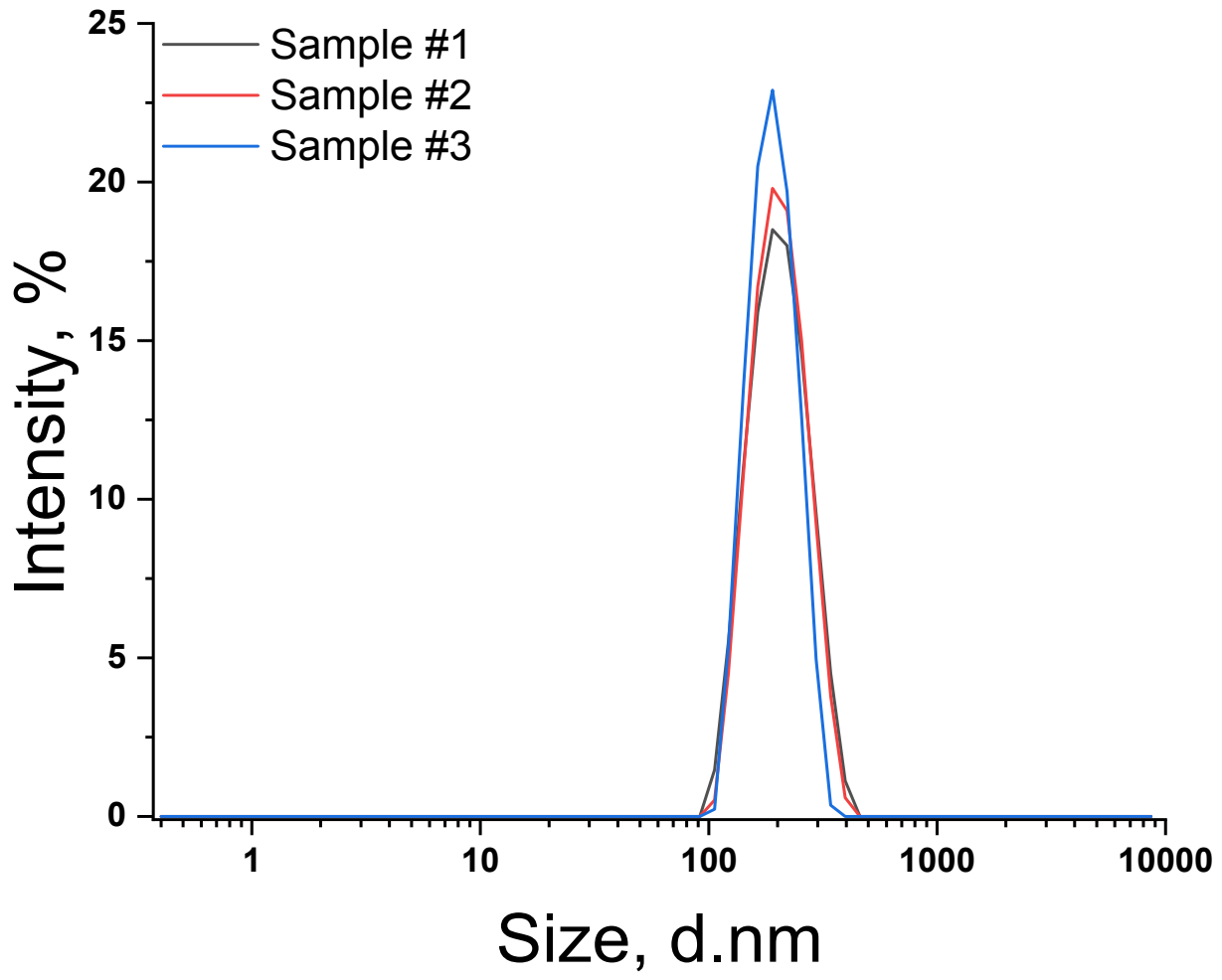


**Figure S1:** DLS results of the average size and size distribution of the nanoparticles.

*Confocal Laser Scanning Microscopy (CLSM)*

Confocal imaging measurements were performed using a Leica TCS 264 SP5 II system (Leica, Germany). Objective HCX PL APO 40.0x0.85 DRY was used for acquisition. Image analysis was performed using LAS X software. The bottoms of Ibidi® μ-Slide 18-well plates were coated with a mixture consisting of 95% vol. silk fibroin solution (5% wt.) and 5% vol. TTA-UC nanocapsule dispersion (10% wt. solid content) and left at 4˚C overnight. Wells were washed with 70% vol. ethanol solution and sterile PBS solution several times in order to sterilize the surface and remove the excess of coating mixture. Images were acquired before and after cell culturing. Fibroblast cells (NIH 3T3, 40 000 per well) were seeded in the already coated Ibidi® wells and incubated overnight in the presence of DMEM medium containing 10% FBS, 1% penicillin/streptomycin in 5% $CO_2$ at 37 ˚C.

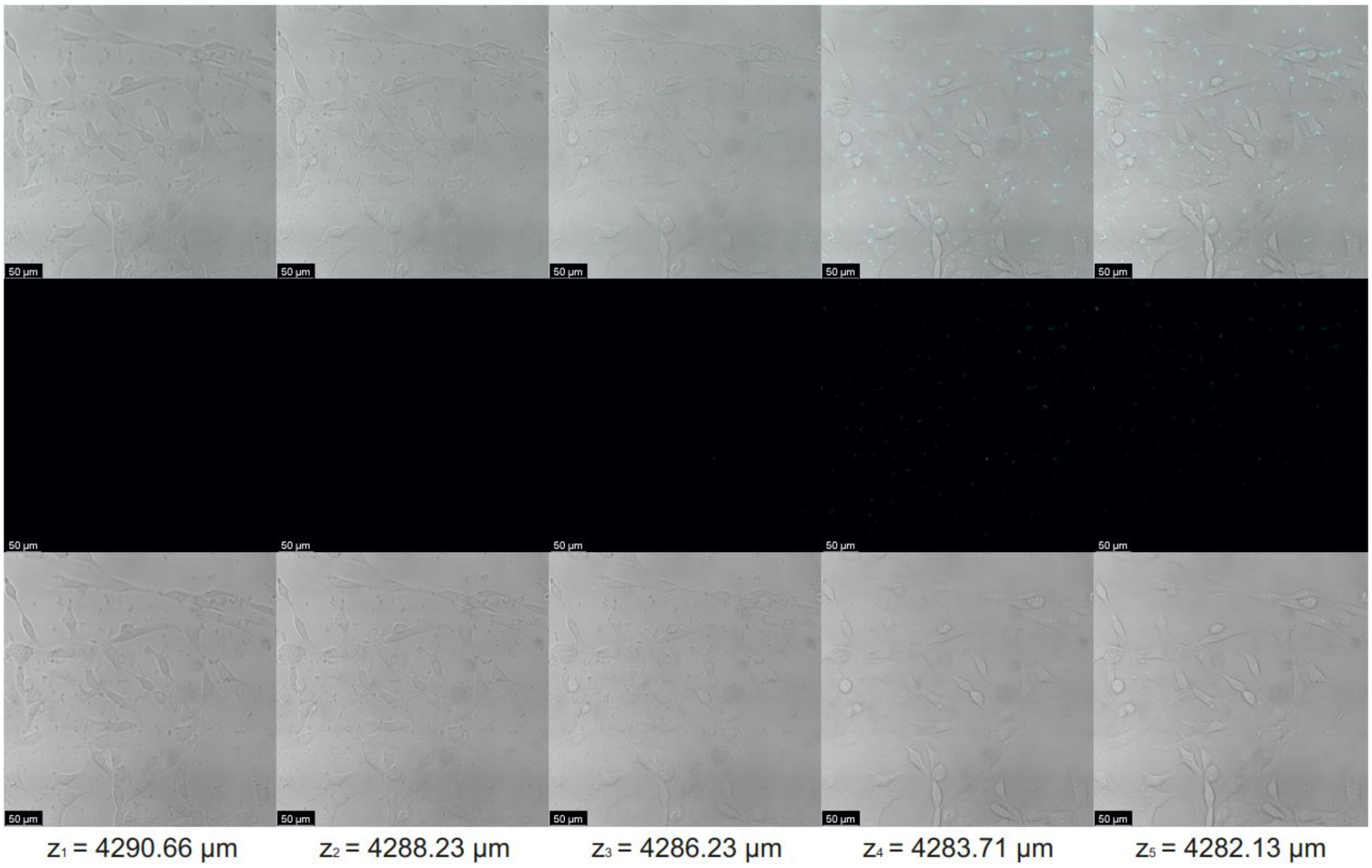


**Figure S2**: Confocal images of NIH 3T3 fibroblast cells seeded on Ibidi® µ-Slide 18-well plates, coated prior with a mixture consisting of 95% vol. silk fibroin solution (5% wt.) and 5% vol. TTA-UC nanocapsule dispersion (10% wt. solid content). From bottom to top: the first image row shows the brightfield image at different z-distances; the second row shows the fluorescence channel; and the third row shows the overlay at different z-distances. The corresponding z-distances are indicated below each image column.

Acquired images show the different z - positions of the sensing-NC and fibroblast cells. The focal plane of the fibroblast cells is located at $z_1$ = 4290.66 µm, whereas the focal plane of the sensing nanocapsules (NCs) is at $z_5$ = 4282.13 µm, indicating that the NCs are located approximately 8 µm below the cells. No cytotoxicity was observed after 24 h. The cells remained viable and adherent, confirming the biocompatibility of the OTAC-stabilized nanocapsules.

*Chemicals*

*Synthesis of PdTBP*

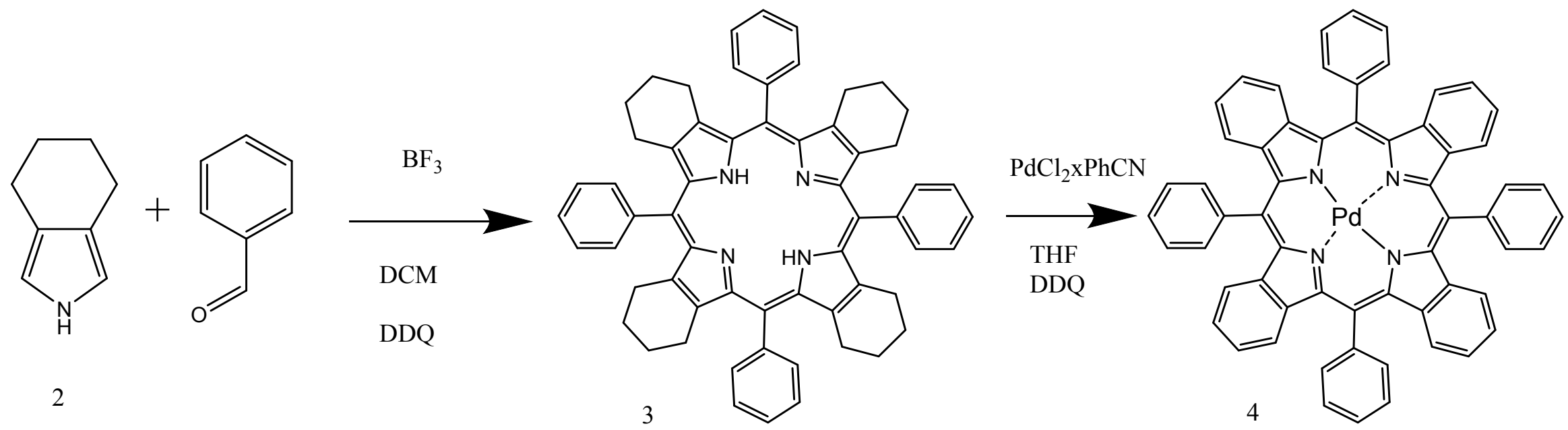


**Figure S3**: Synthesis of PdTBP.

**Tetraphenyltetrabenzoporphyrin 3:** Tetrahydroisoindole **2** (144 mg, 0.118 mmol, 1.0 equiv) was dissolved in dry DCM (dichloromethane) (100 mL). The reaction mixture was degassed for 20 min with argon. The stirred mixture was protected from light and stirred under argon. Benzaldehyde (125 mg, 0.118 mmol, 1.0 eq.) was added, and the reaction mixture was kept in the dark under argon and stirred for 10 min. $BF_3$ $Et_2O$ (0.7 mL of 1% solution in $CH_2Cl_2$) was added in one portion, and the mixture was allowed to react at room temperature for 4 h. DDQ (2,3-dichloro-5,6-dicyano-1,4-benzoquinone) (110 mg, 41.8 mmol, 5.0 eq.) was added to the mixture in one portion, and the mixture was stirred at room temperature for 2 h. The resulting mixture was washed with 10% aqueous $Na_2SO_3$ (2x100 mL) and with water (2x100 mL). The combined organic phases were dried over $Na_2SO_4$. The solvent was evaporated, and the resulting residue was crystallized from methanol-DCM mixture. It was used in the next step without chromatographic purification.

**PdTBP 4:** An excess of $PdCl_2(PhCN)_2$ (140 mg, 0.36 mmol, 3.0 equiv.) was added to a solution of **3** (100 mg, 0.12 mmol, 1.0 equiv.) in THF (Tetrahydrofuran) (40 mL) with triethylamine (2 mL), and the mixture was refluxed for 2 h. DDQ (210 mg, 0.94 mmol, 10 equiv.) was added, and the mixture was refluxed for 1 h. The solvent volume was reduced to 3 mL under vacuum. DCM (100 mL) was added, and the mixture was washed with aqueous 10% $Na_2SO_3$. The organic phase was dried over $Na_2SO_4$, and the solvent was evaporated. The crude product was

purified by column chromatography on silica gel (eluent: toluene). Pure **4** was isolated as a dark-green solid with an overall yield of 70 mg (63%). $^{1}$H-NMR ($CDCl_3$, 700 MHz) δ H = 8.27 (8H, dd, $^{3}$J = 7.5, $^{4}$J = 1.5), 7.92 (4H, tt, 3J = 7.5, 4J = 1.5), 7.85 (8H, dd, 3J = 7.5, 4J = 1.5), 7.21 (8H, dd, 3 J = 6.5, 4J = 3.0), 7.12 (8H, dd, 3J = 6.5, 4J = 3.0). $^{13}$C-NMR ($CDCl_3$, 176 MHz) δ C = 142.4, 139.1, 138.5, 134.5, 129.7, 129.6, 125.8, 124.4, 118.6. MS (MALDI, dithranol matrix): m/z = 917.4 [M] + ; calculated for $C_{60}H_{36}N_4Pd$ 918.20.

## 2. Figures and Data

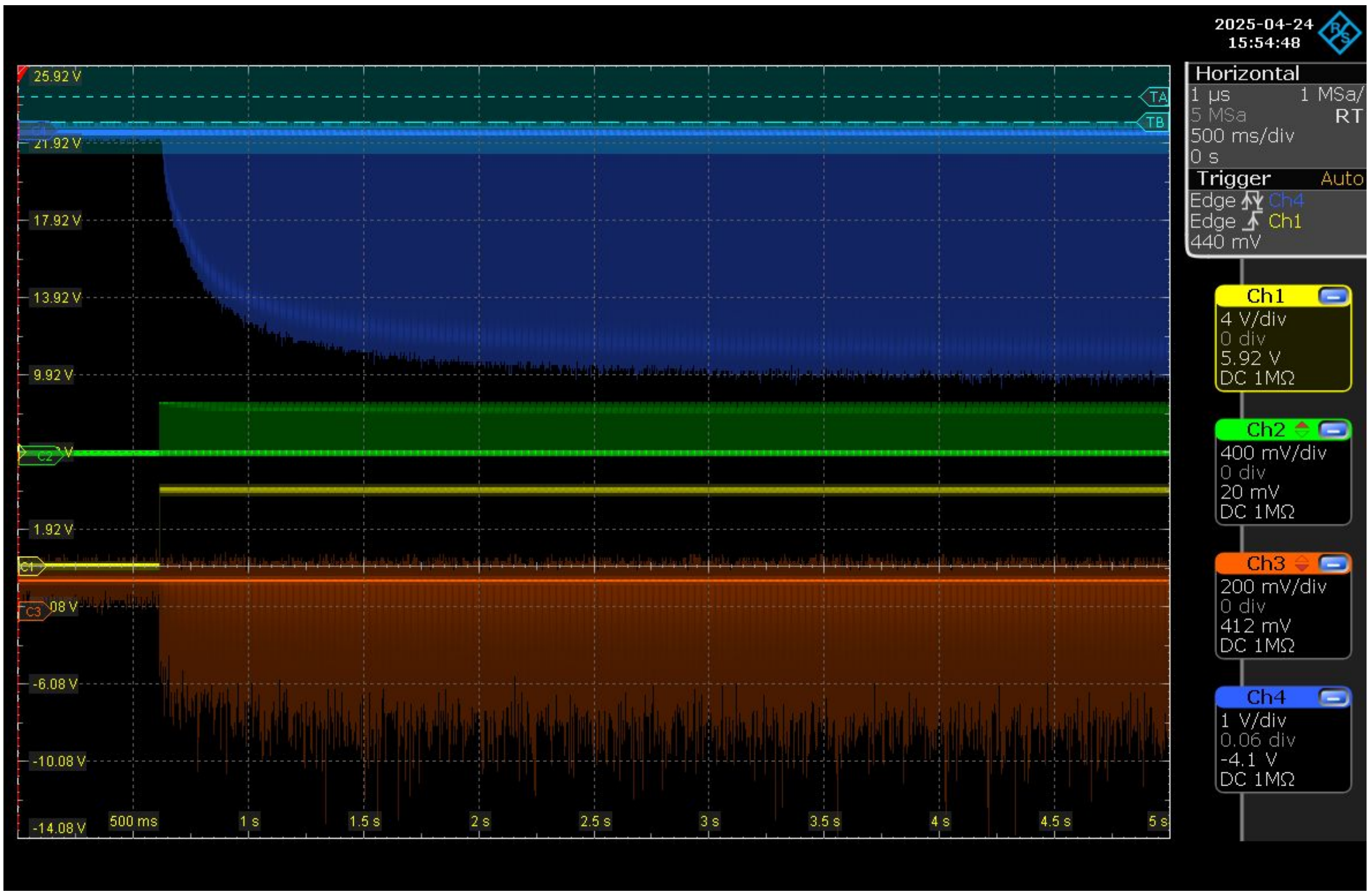


**Figure S4**: Time-resolved intensity of the optical signals of dF (the blue channel), rPh (the orange channel) for the TTA-UC molecular system (presented in Figure 1) together with the intensity of the excitation laser pulse (the green channel) and measurement start (the yellow channel). Nanoparticle architecture and materials composition is shown in Figure 2. ***Experimental Conditions***: TTA-UC NC dispersed in water, placed in ambient environment (160 mmHg $O_2$ partial pressure, corresponding to normoxia); sample temperature = 24 ˚C; sample thickness d = 1×10$^{-3}$ m; NC – solid content 5% wt.; peak excitation intensity $I_{exc}$ = 6.6

W×cm$^{-2}$; wavelength $\lambda = 631$ nm; excitation pulse duration $\tau_{pulse}$ = 1.5 ms; excitation pulse period $\tau_{pulse}$ = 6 ms; observation duration $t_{sensing}$ = 4.8 s (800 successive excitation pulses); acquisition time resolution $\tau_{acquisition} = 1$ µs.

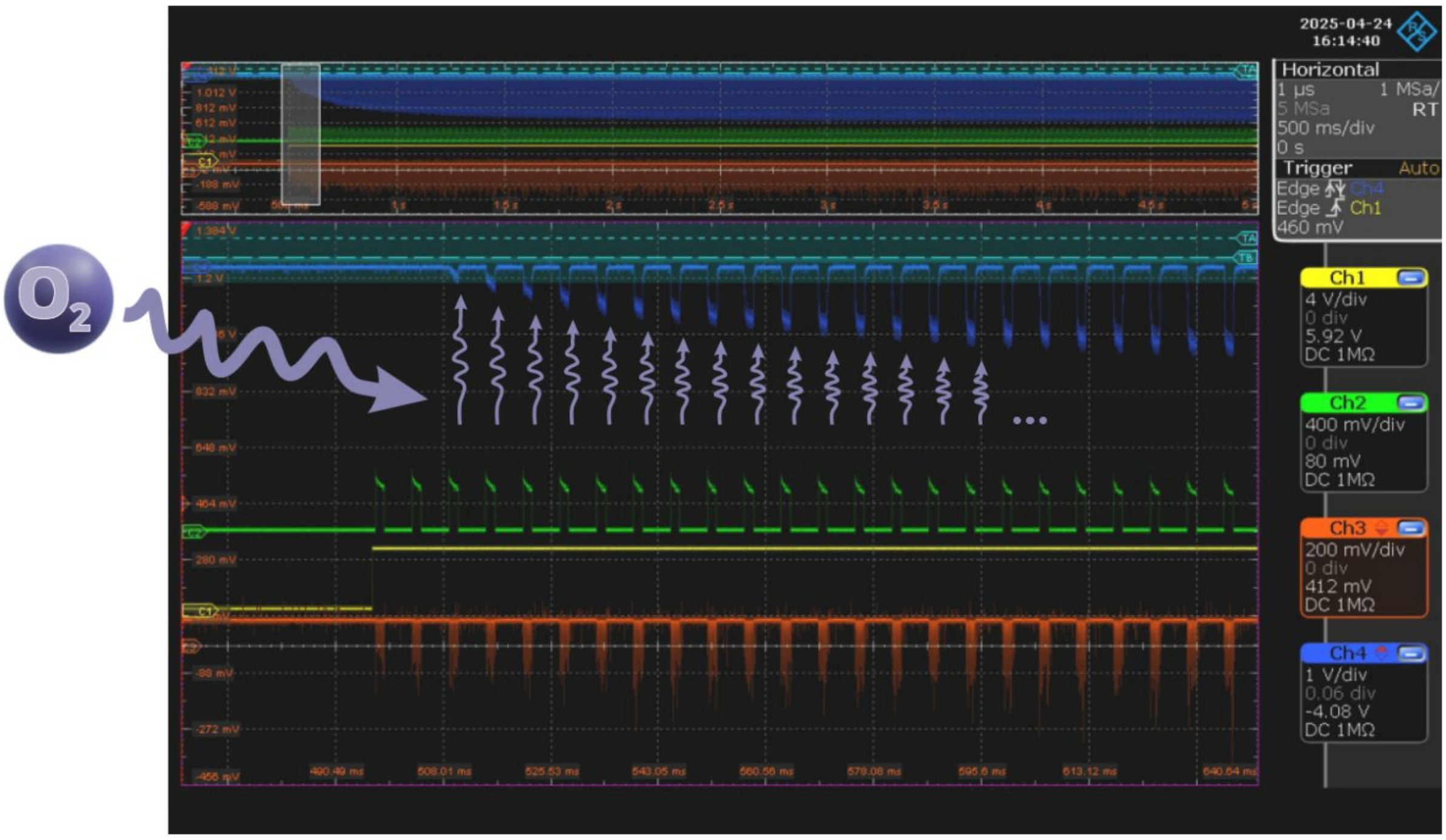


**Figure S5**: Time-resolved intensity of the optical signals of dF (the blue line) and rPh (the red line) for the TTA-UC system, presented in Figure 1: magnified are the first 25 pulses. ***Experimental Conditions***: the same as in Figure S4.

## Laser setup

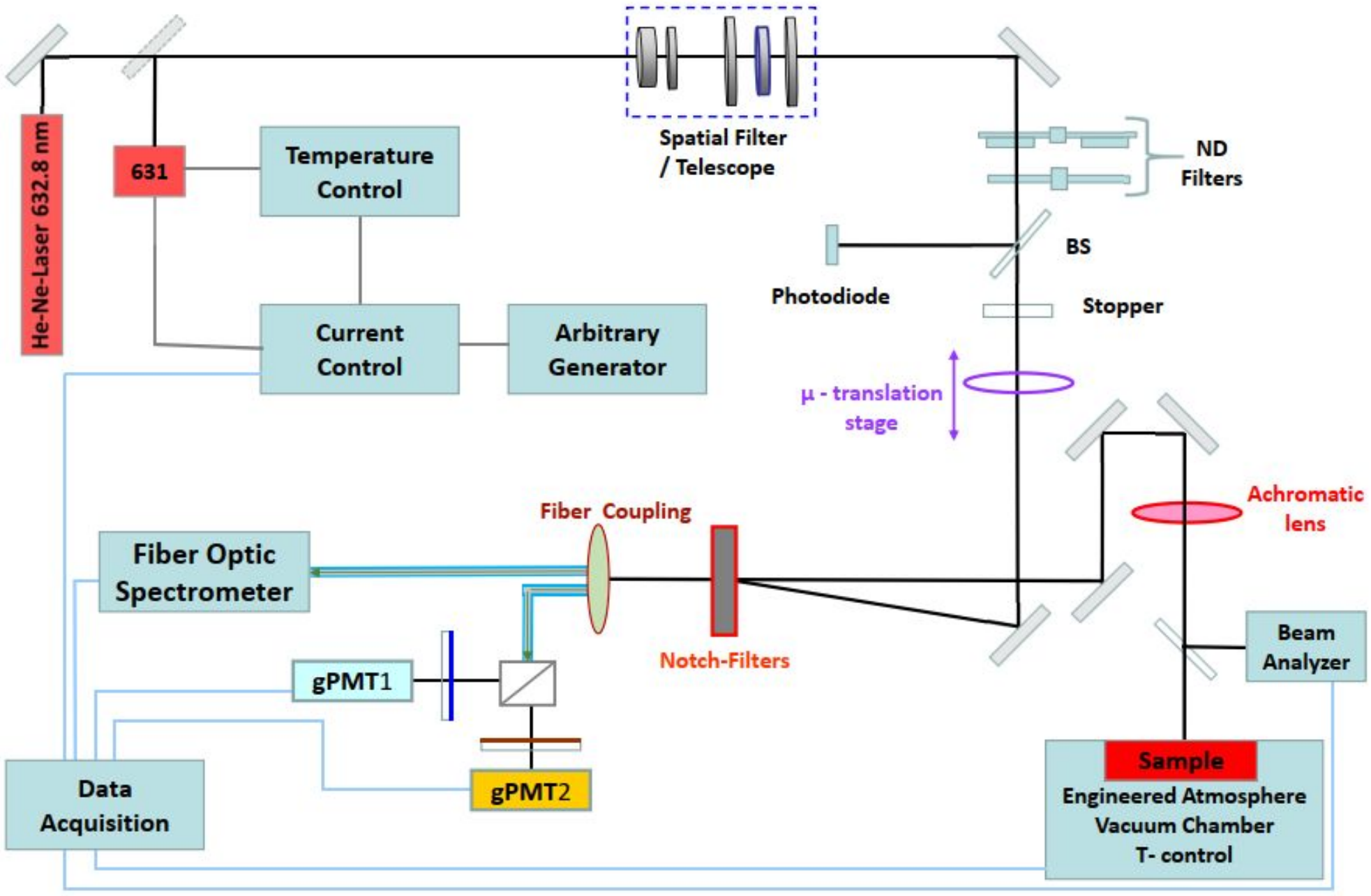


**Figure S6**: Set-up for registration of the dynamical parameters of the TTA-UC in engineered atmosphere with actively controlled oxygen content, active temperature control and temperature stabilization. In order to minimize the effect of solvent convection, the liquid samples are measured in horizontal position.

The diode laser beam pass through the spatial filter in order to reach nearly TEM00 transversal intensity distribution. A series of reflective neutral density (ND) filter (Thorlabs Inc.) placed on a revolving optical holder (in order to keep the optical axes of the experiment unchanged) were used to attenuate smoothly the beam power. Afterward, the beam passed through the system of ultra-broadband mirrors (MaxMirror, *Semrock Inc.*) and finally focused by achromatic lens (NA = 0.24) onto the sample. A defocusing achromatic lens (the violet lens) placed on an electronically controlled µ-positioning stage (DL125 Delay Line Stage, *Newport Corp.*) controls the excitation spot diameter. The resulting excitation spot diameter is permanently controlled by a beam profiler (BP104-VIS, *Thorlabs Inc.*). The optical density of all samples at the excitation wavelength is nearly 0.1; therefore, the attenuation of the pump light can be neglected for the given thickness of the optical samples (1000 µm or 400 µm). The luminescence emission generated by the sample was collected with the same apochromatic lens, thus the excitation- and observation- spots are completely spatially overlapped. The

emission of the excitation lasers was rejected by the appropriate notch filters: for instance by application of a notch filter designed for $\lambda = 633$ nm (FWHM ~ 29 nm, NF 03-633E-25, *Semrock Inc.*) rejection better than $10^6$ times can be achieved.

The *cw*-emission spectra were registered by Optical Multichannel Analyzer (*Hamamatsu Photonics Inc.*) or fiber – spectrometer (C10083CA, *Hamamatsu Photonics Inc.*) with absolute wavelength calibration and corrected spectral response.

For the time-resolved measurements of the sensitizer residual phosphorescence and delayed emitter fluorescence, additional removable beam splitters were installed in order to reflect the sample emission toward the time-resolved registration unit. The used laser heads (TCLDM9, *Thorlabs Inc.*) allow current modulation *via* modulation input, depending on the maximal current supplied, the modulation frequency varies from 50 kHz up to 200 kHz. Therefore, the laser intensity was modulated by an arbitrary pulse generator (TGA1244, *TTi Inc.*) with caring frequency of 40 MHz. The obtained laser diode pulses have almost rectangular time-shape, with rise- and decay- slopes less than $2.5\times10^{-6}$ s.

One edge-beam splitter (FF01-694/SP-25, *Semrock Inc.*) was used to spatially separate the sensitizer residual phosphorescence and delayed emitter UC-fluorescence, registered by gPMT1 and gPMT2, accordingly. Additional edge-filters, namely a short pass (SP01-633RS-25, *Semrock Inc.*) filter with cutting wavelength $\lambda = 650$ nm and long pass (HQ700LP, *Chroma GmbH*) filter with cutting wavelength $\lambda = 700$ nm, were installed in front of the registration opto-electronic devices at the delayed-fluorescence branch and phosphorescence branch, respectively.

The optical signals were detected by gated photo-multiplier tube gPMT1 (H1156-01-NF, *Hamamatsu Photonics Inc.*) and gated photo-multiplier tube gPMT2 (H1156-20-NF *Hamamatsu Photonics Inc.*, with extended red multialkali photocathode with enhanced sensitivity). The gPMT's are working in analogous mode with a rise- and decay- times less

than 30×10$^{-9}$ s. The gated photo-multiplier tubes are set in "Normally OFF" mode. The gating pulses should be with an amplitude of 3.5 V ÷ 5 V applied on 10 kΩ load, with maximal repetition rate of 10 kHz. The gPMT-operation delay is less than 0.2×10$^{-6}$ s. Each gPMT is equipped with a wide bandwidth amplifier unit C9663 (DC up to 150MHz, *Hamamatsu Photonics Inc.*), where the current input signal from the photomultiplier tube is directly converted into a voltage output signal.

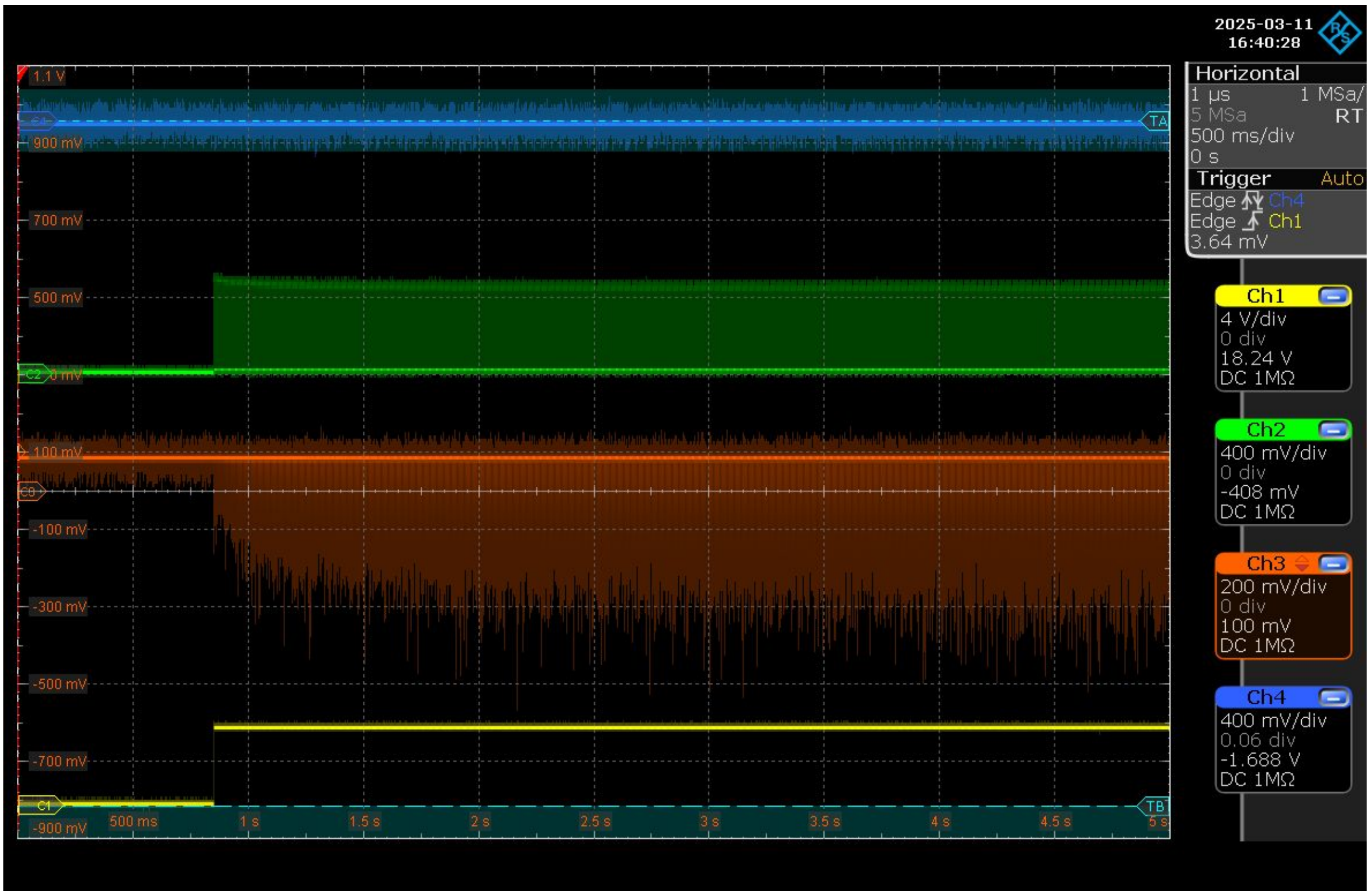


**Figure S7**: Time-resolved intensity of the optical signals of Ph (the orange channel) for the TTA-UC molecular system (presented in Figure 1) together with the intensity of the excitation laser pulse (the green channel) and measurement start (the yellow channel). Nanoparticle architecture and materials composition is shown in Figure 2 with the exception, that no emitter molecules are present. ***Experimental Conditions***: NC dispersed in water, placed in ambient environment (160 mmHg $O_2$ partial pressure, corresponding to normoxia); sample temperature $T$ = 24 °C; sample thickness $d$ = 1×10$^{-3}$ m; NC – solid content 5% wt.; peak excitation intensity

$I_{exc}$ = 6.6 W×cm$^{-2}$; wavelength $\lambda$ = 631 nm; excitation pulse duration $\tau_{pulse}$ = 1.5 ms; excitation pulse period $\tau_{pulse}$ = 6 ms; observation duration $t_{sensing}$ = 4.8 s (800 successive excitation pulses); acquisition time resolution $\tau_{acquisition}$ = 1 µs.

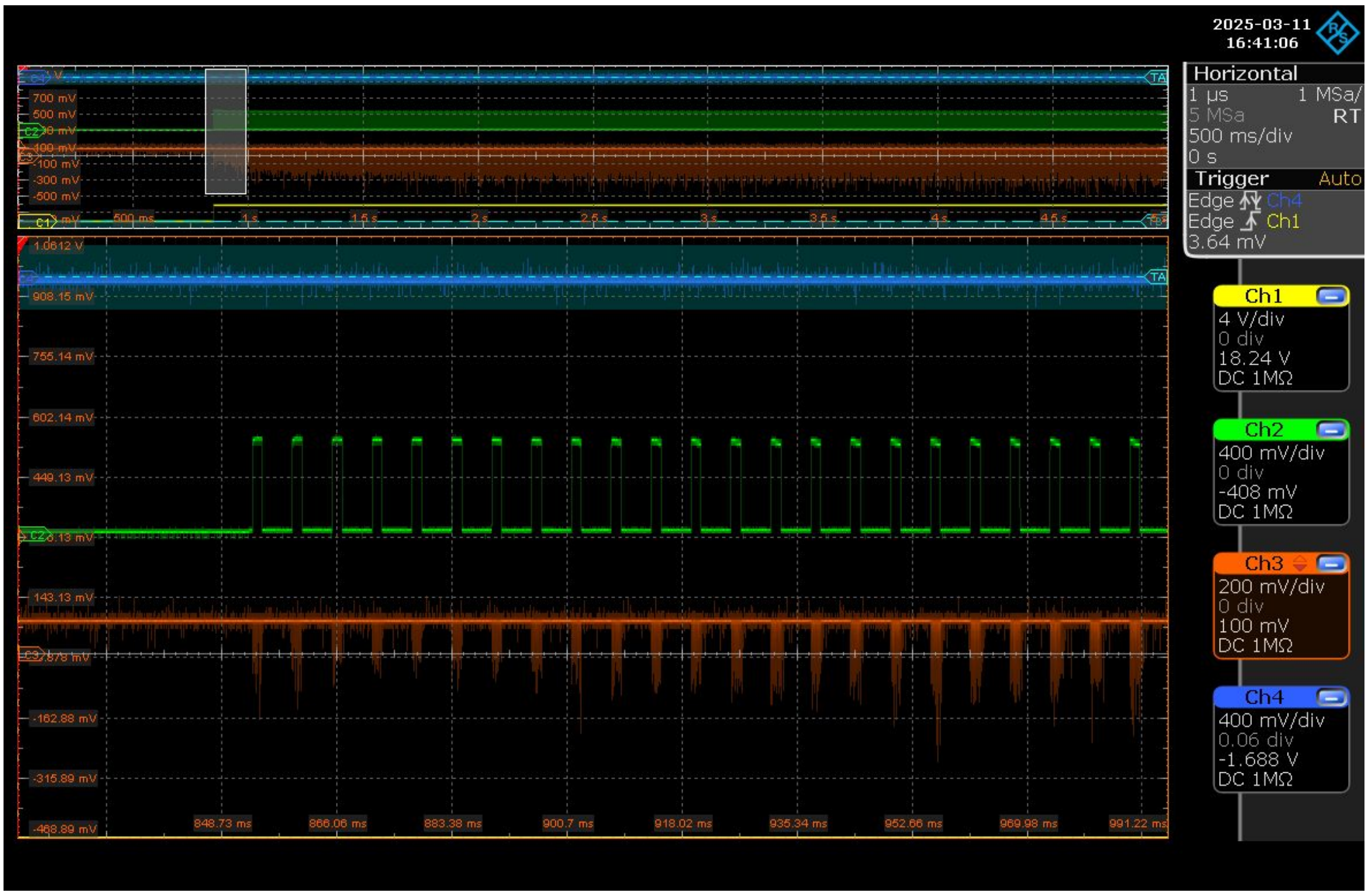


**Figure S8**: Time-resolved intensity of the optical signals of dF (the blue line) and rPh (the red line) for the TTA-UC system, presented in Figure 1: magnified are the first 25 pulses. ***Experimental Conditions***: the same as in Figure S7.

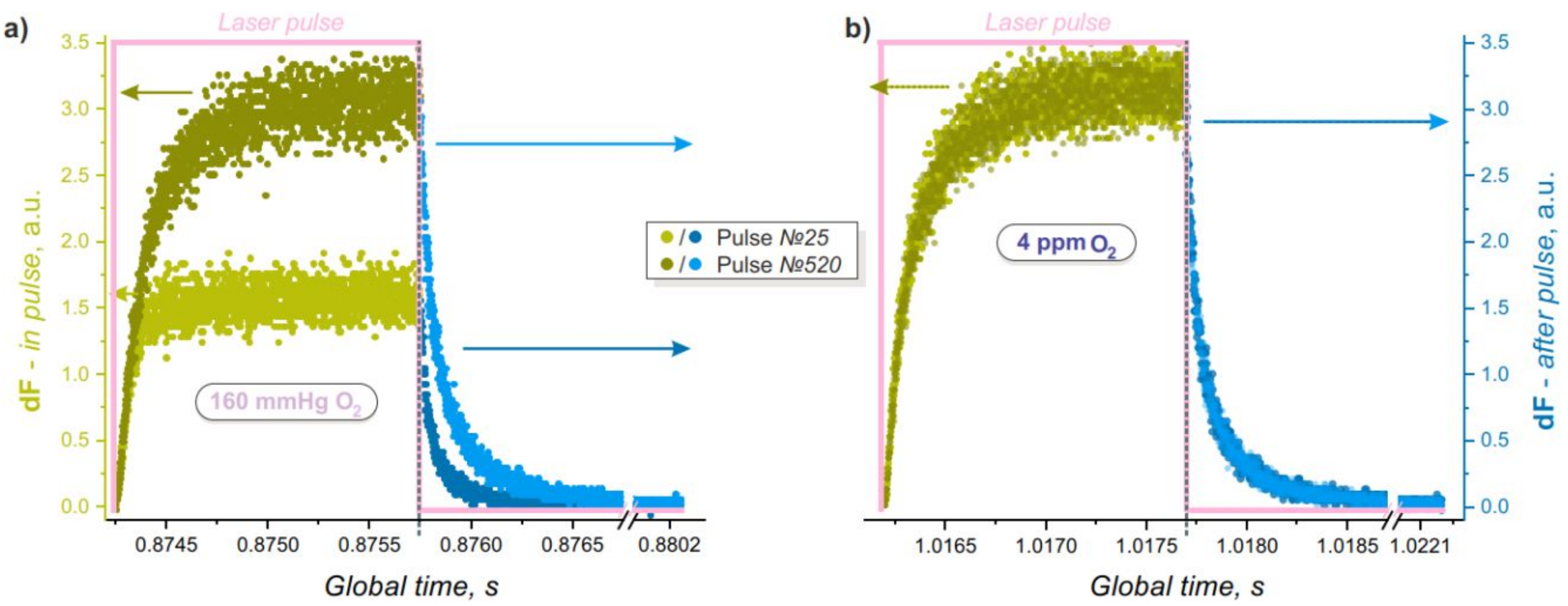

**Figure S9**: Time-resolved intensity of the optical signals of dF for the TTA-UC molecular system (presented in Figure 1) at two environmental boundary conditions – (a) normoxia and (b) deep anoxia. Compared are the dF signals, first – 150 ms after excitation start (pulse N25) and 3120 ms after excitation start (pulse N520). ***Experimental Conditions***: Nanocapsule architecture and materials composition is shown in graphical abstract; TTA-UC NC dispersed in water; sample temperature = 24 ˚C; sample thickness d = $1\times10^{-3}$ m; NC – solid content 5% wt.; peak excitation intensity $I_{exc}$ = 6.6 W×cm$^{-2}$; wavelength $\lambda = 631$ nm; excitation pulse duration $\tau_{pulse} = 1.5$ ms; excitation pulse period $\tau_{pulse} = 6$ ms; observation duration $t_{sensing}$ = 4.8 s (800 successive excitation pulses); acquisition time resolution $\tau_{acquisition} = 1$ µs.

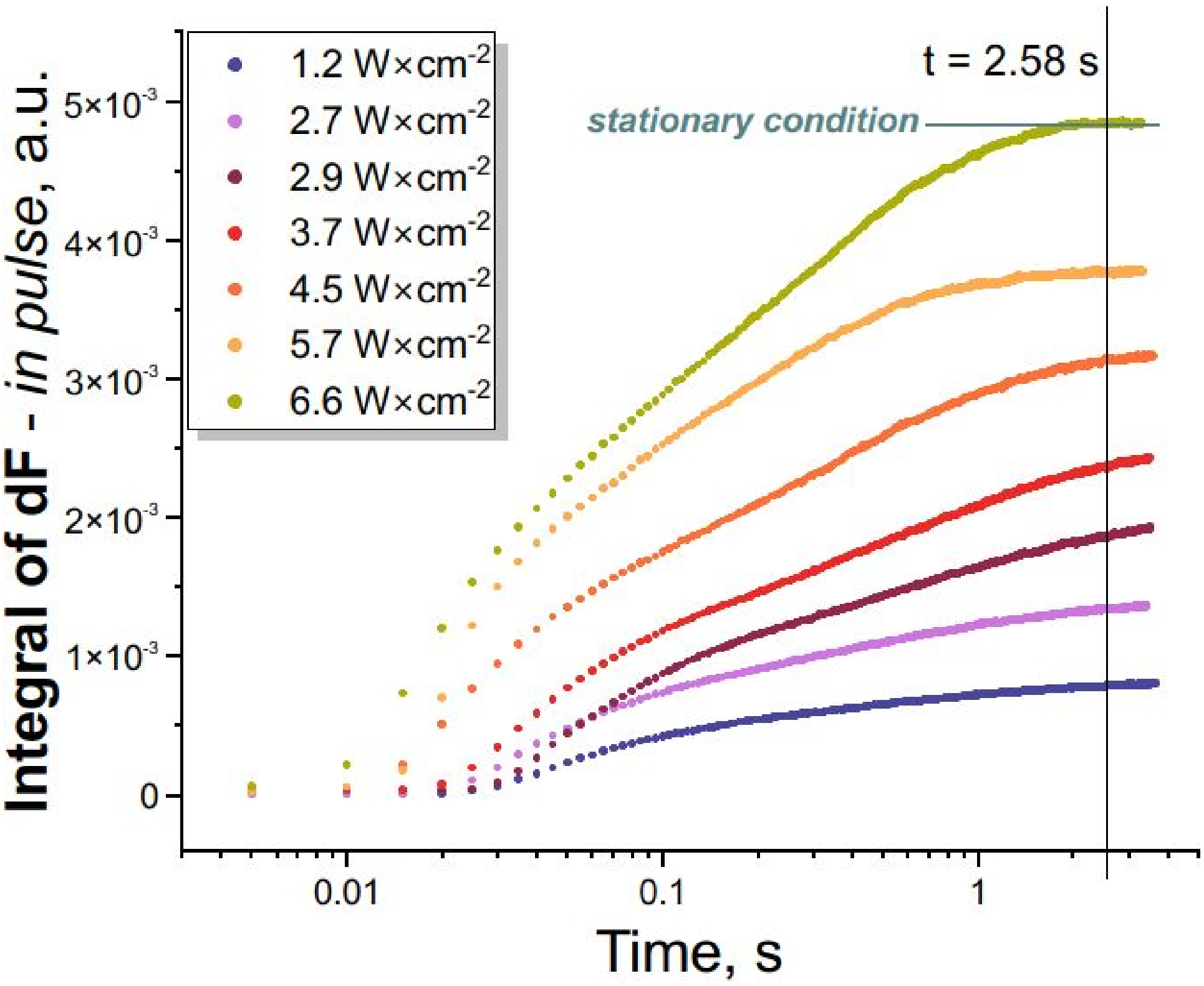


**Figure S10**: Dependence of $I_{in}^{dF}(n)$ signal for different peak excitation intensities, shown in the figure. The excitation pulse durations ($\tau_{pulse}$ = 1.5 ms) and the excitation pulse periods for all measurements are identical – $\tau_{pp}$= 6 ms. ***Experimental Conditions***: the same as in Figure S4.

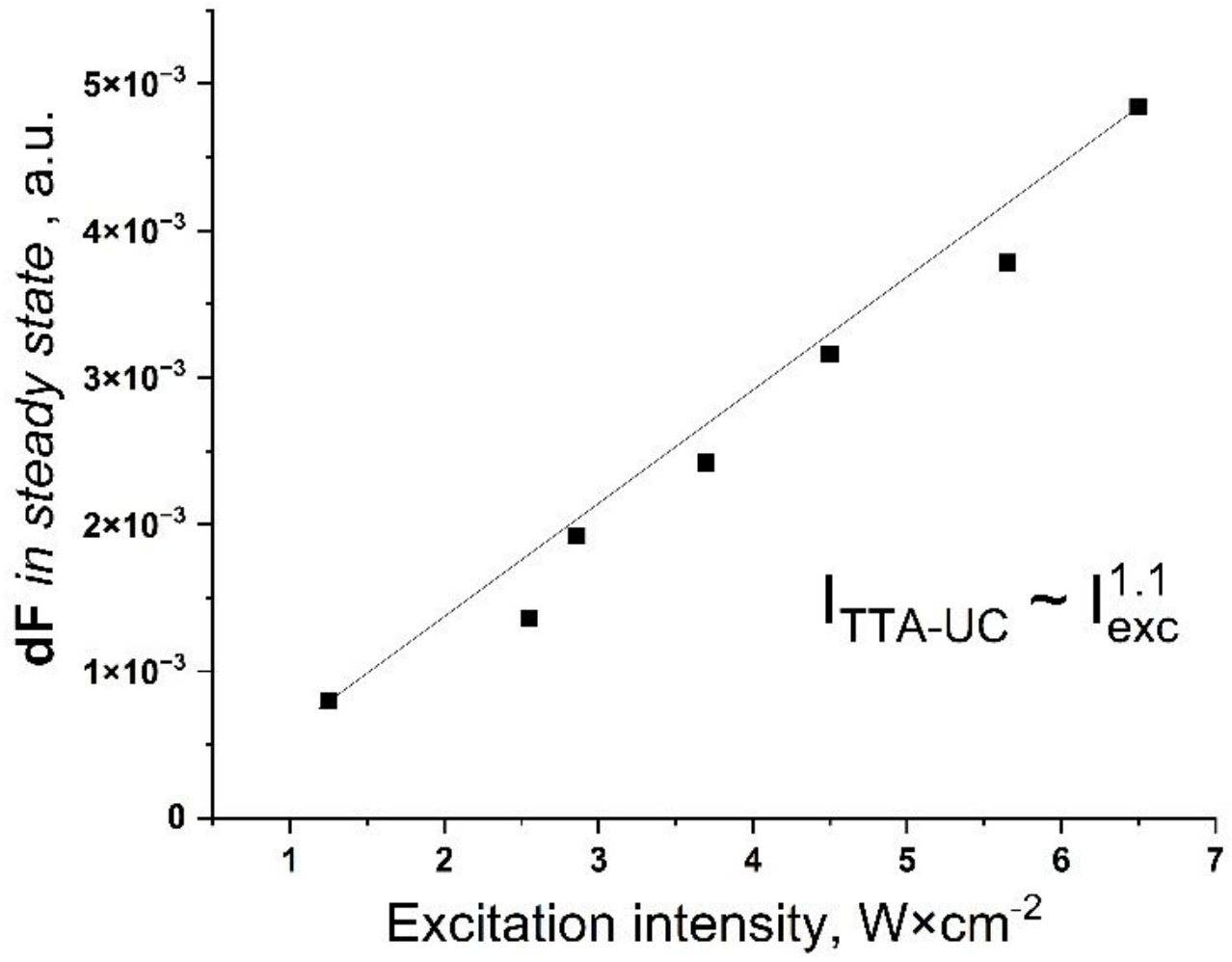


**Figure S11**: Dependence of the dF on excitation intensity, after reaching the steady-state emission under constant optical excitation. ***Experimental Conditions***: TTA-UC NC dispersed in water, placed in ambient environment (160 mmHg $O_2$ partial pressure, corresponding to normoxia); sample temperature = 24 °C; sample thickness $d = 1\times10^{-3}$ m; NC – solid content 5% wt.; wavelength $\lambda = 631$ nm; Excitation intensity - shown in the graph.

As shown in Figure S11, even after reaching a steady-state dF emission under constant optical excitation, the intensity dependence (of the nanoconfined TTA-UC system) is super-linear: the experimental data are well approximated with a power law $I_{TTA-UC} = a \times I_{exc}^{b}$, where the parameter $\boldsymbol{b} = 1.11$. This super-linear intensity dependence is demonstrated for many energetically optimized TTA-UC molecular couples emitter / sensitizer.[1]

The delayed fluorescence (dF), resulting from the process of nanoconfined TTA-UC in oxygen rich environment does not obey the Stern–Volmer equation directly, because oxygen quenches the sensitizer triplet population, not the emitted fluorescence itself. The dynamical properties of dF are therefore a consequence of complex dynamics of the sensitizer triplet concentration.

Further, the classical representations of the Stern–Volmer dynamics assume that the resulting dF intensity depends either quadratically[2] or linearly[3] on the excitation optical intensity.

Furthermore, the global luminescence loss, $L_{pO_2}^{dF}$ reported in this study exhibits a one-to-one, correlation with the local oxygen concentration when the dependence of dF on the local oxygen

concentration is determined in the so-called non-equilibrium regime, i.e., before the dF emission reaches a steady-state value under constant optical excitation. During this initial excitation period, the intensity dependence of the dF cannot be reliably determined.

The TTA-UC process is diffusion-controlled, with its kinetics determined by the diffusion of molecular oxygen within the NC core, the diffusion of molecular oxygen across the NC shell, and the scavenging rate of the generated singlet oxygen through photooxidation of the SSOS material. It is important to note that the quencher concentration cannot be described by the classical model without accounting for quencher instantaneous depletion resulting from its chemical reaction with the scavenging molecules.

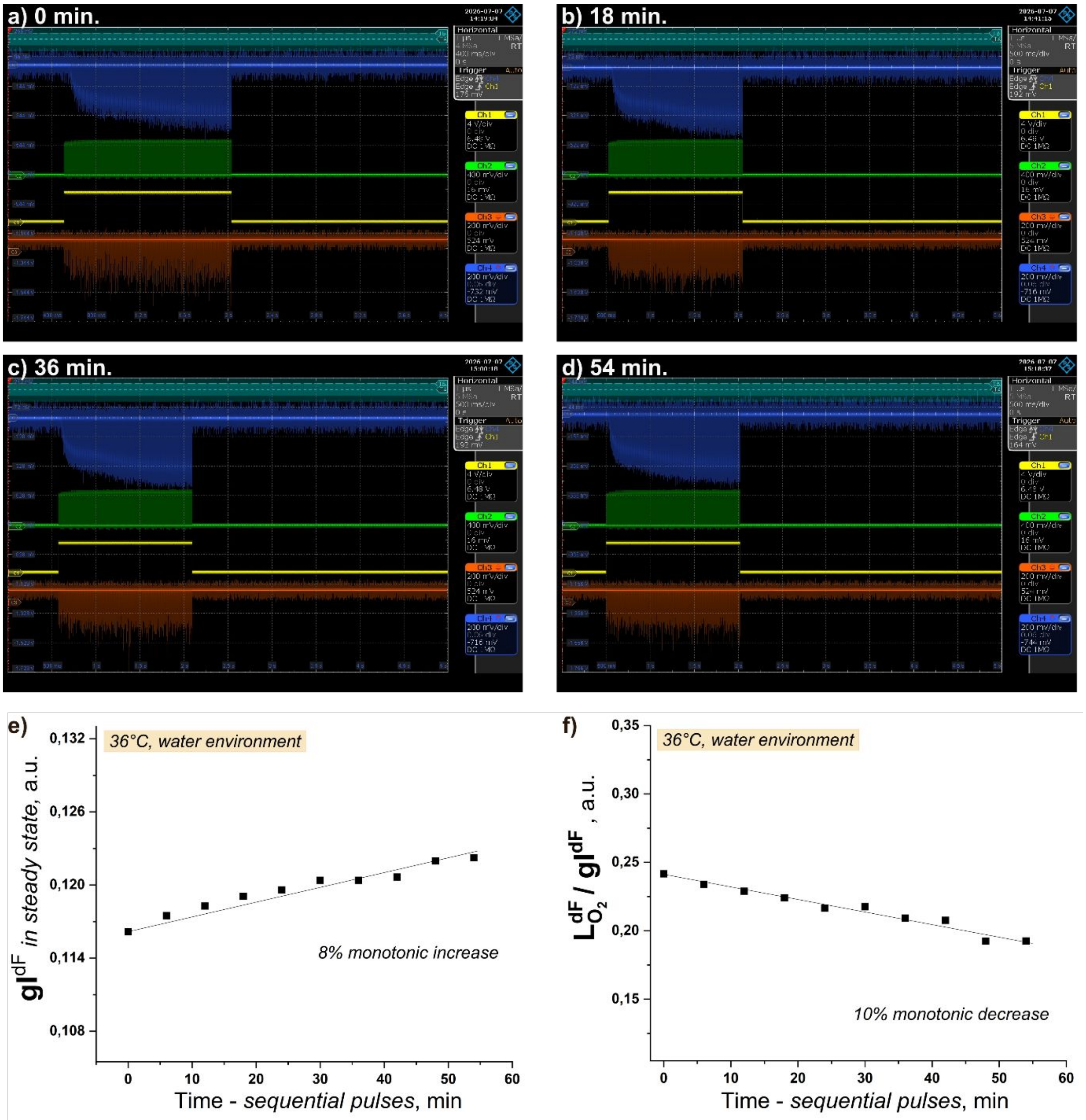


**Figure S12**: (**a**) – (**d**) Time-resolved intensity of the optical signals of dF (the blue channel), rPh (the orange channel) for the TTA-UC molecular system (presented in Figure 1) together with the intensity of the excitation laser pulse (the green channel) and measurement start (the yellow channel) for sequential excitation pulses (acting at the same spot) ; (**e**) – Dependence of the global delayed fluorescence intensity, $gI^{dF}$ (measured at steady state in the oxygen-free NC core) on the excitation sequence number; (**f**) – Dependence of the ratio of the global luminescence loss, $L_{pO_2}^{dF}$ (at a given oxygen concentration) to the global delayed fluorescence intensity $gI^{dF}$ on the excitation sequence number. ***Experimental Conditions***: Time span

between the sequential excitation pulses, $t_{seq}$ = 6 min; water solution of NC (solid content 2.5% wt.; sample temperature = 36 ˚C; sample thickness d = 1×10$^{-3}$ m; wavelength $\lambda$ = 631 nm; excitation pulse duration $\tau_{pulse}$ = 1.5 ms; excitation pulse period $\tau_{pp}$ = 6 ms; observation duration $t_{sensing}$ = 2.796 s (466 successive excitation pulses); acquisition time resolution $\tau_{acquisition}$ = 1 μs. TTA-UC NC dispersed in water mixtures (described below), placed in ambient environment (160 mmHg $O_2$ partial pressure, corresponding to normoxia); Nanoparticle architecture and materials composition is shown in Figure 2; peak excitation intensity $I_{exc}$ = 1.4 W×cm$^{-2}$. ***Time sequence***: (**a**) – sequence 1 (t = 0); (**b**) – sequence 4 (*t* = 18 min.); (**c**) – sequence 7 (*t* = 36 min.); (**d**) – sequence 10 (t = 54 min.).

As shown in Figure S12 a–e, the absolute intensity of the global delayed fluorescence, g$I^{dF}$ , measured after reaching the steady state, increases monotonically with the number of excitation pulses. The stationary conditions are reached at *t* = 2.48 s. For the given NC composition, this monotonic increase is less than 8% (Figure S12 e), indicating that the dark period should be extended to allow complete recovery of the oxygen content within the NC between successive excitation cycles. This result unequivocally demonstrates that the $L_{pO_2}^{dF}$ – oxygen calibration curves can be tuned in a controlled and readily automatable manner, provided that the excitation sequence number is known (Figure S12 f).

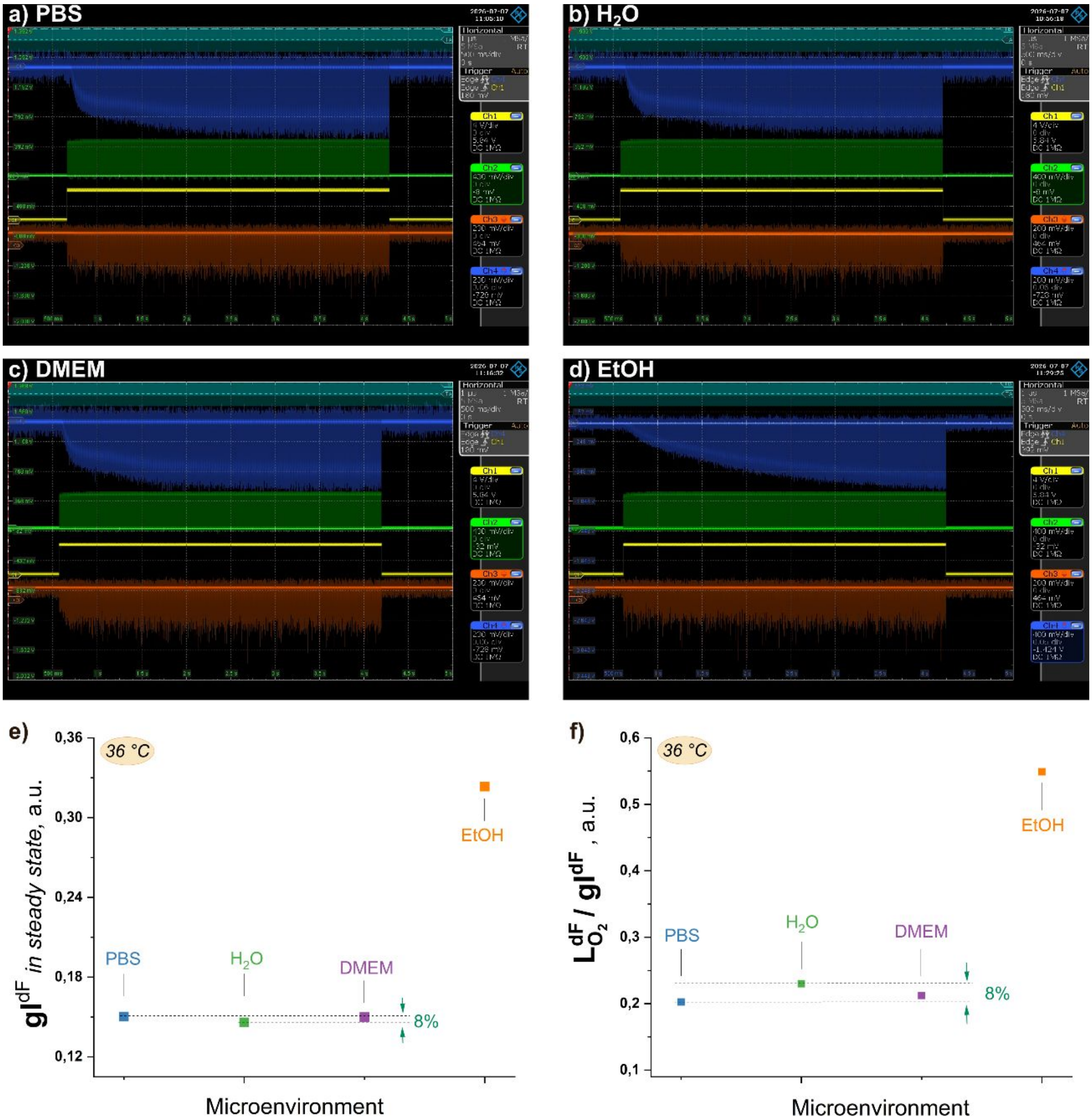


**Figure S13**: (**a**) – (**d**) Time-resolved intensity of the optical signals of dF (the blue channel), rPh (the orange channel) for the TTA-UC molecular system (presented in Figure 1) together with the intensity of the excitation laser pulse (the green channel) and measurement start (the yellow channel) for different composition of the continuous phase; (**e**) – Dependence of the global delayed fluorescence intensity, $gI^{dF}$ (measured at steady state in the oxygen-free NC core) on the composition of the aqueous microenvironment; (**f**) – Dependence of the ratio of the global luminescence loss, $L^{dF}_{pO_2}$ (at a given oxygen concentration) to the global delayed fluorescence intensity $gI^{dF}$ on the composition of the aqueous microenvironment.

***Experimental Conditions***: Sample temperature = 36 ˚C; sample thickness d = 1×10$^{-3}$ m; wavelength λ = 631 nm; excitation pulse duration $\tau_{pulse}$ = 1.5 ms; excitation pulse period $\tau_{pp}$ = 6

ms; observation duration $t_{sensing}$ = 4.8 s (800 successive excitation pulses); acquisition time resolution $\tau_{acquisition}$ = 1 µs. TTA-UC NC dispersed in water mixtures (described below), placed in ambient environment (160 mmHg $O_2$ partial pressure, corresponding to normoxia); Nanoparticle architecture and materials composition is shown in Figure 2; peak excitation intensity $I_{exc}$ = 1.4 W×cm$^{-2}$.

***Sample compositions***: (**a**) - water mixture of 50% vol. NC (solid content 5% wt.) and 50% vol. PBS (concentration 2×); (**b**) – water solution of NC (solid content 2.5% wt.); (**c**) – water mixture of 50% vol. NC (solid content 5% wt.) and 50% DMEM; (**d**) - water mixture of 50% vol. NC (solid content 5% wt.) and 50% vol. EtOH (Ethanol absolute food grade 99.8% vol., Sigma Aldrich, CAS 64-17-5).

As shown in Figure S13 (a, b and c), partial replacement of the continuous water phase with PBS, DMEM, or pure water, while maintaining a constant NC solid content, does not significantly alter the TTA-UC response. The stationary conditions are reached at $t$ = 2.48 s. for the sample containing water and NC. In contrast, a substantially different response is observed when the total ethanol (EtOH) concentration is increased to levels sufficient to eliminate biological contamination (Figure S13d). Nevertheless, exposure to such EtOH concentrations will be limited to a short duration, followed by washing of the hydrogels incorporating the sensing NC.

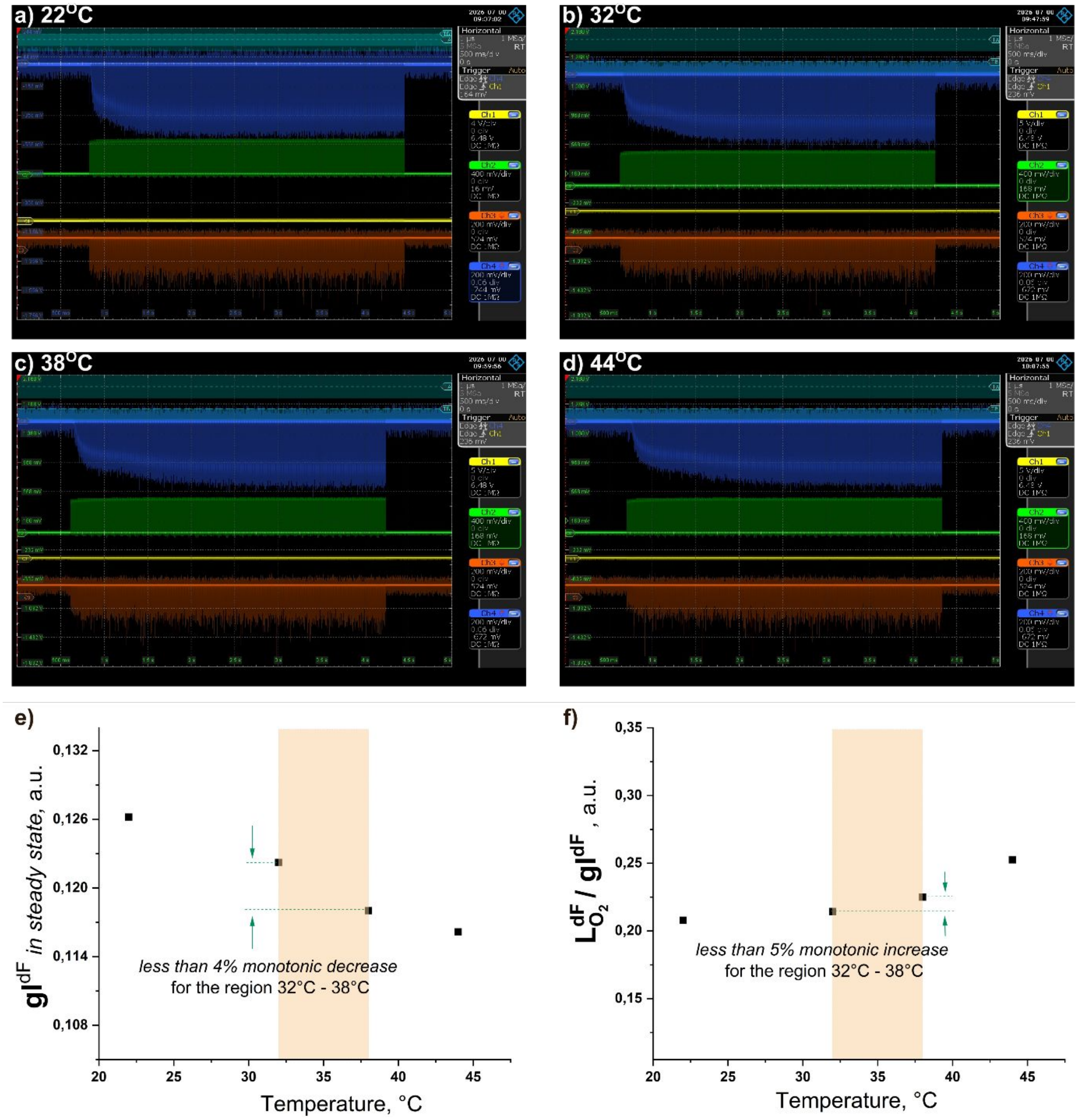


**Figure S14**: (**a**) – (**d**) Time-resolved intensity of the optical signals of dF (the blue channel), rPh (the orange channel) for the TTA-UC molecular system together with the intensity of the excitation laser pulse (the green channel) and measurement start (the yellow channel). for different sample temperatures; (**e**) – Dependence of the global delayed fluorescence intensity, $gI^{dF}$ (measured at steady state in the oxygen-free NC core) on the temperature of aqueous microenvironment; (**f**) – Dependence of the ratio of the global luminescence loss, $L^{dF}_{pO_2}$ (at a

given oxygen concentration) to the global delayed fluorescence intensity $gI^{dF}$ on the temperature of the aqueous microenvironment.

***Experimental Conditions***: Water solution of NC (solid content 2.5% wt.); Sample thickness d = $1\times10^{-3}$ m; wavelength $\lambda$ = 631 nm; excitation pulse duration $\tau_{pulse}$ = 1.5 ms; excitation pulse period $\tau_{pp}$ = 6 ms; observation duration $t_{sensing}$ = 4.8 s (800 successive excitation pulses); acquisition time resolution $\tau_{acquisition}$ = 1 µs. TTA-UC NC dispersed in water mixtures (described below), placed in ambient environment (160 mmHg $O_2$ partial pressure, corresponding to normoxia); Nanoparticle architecture and materials composition is shown in Figure 2; peak excitation intensity $I_{exc}$ = 1.4 W×cm$^{-2}$. ***Sample temperatures***: (**a**) – 22 ˚C; (**b**) – 32 ˚C; (**c**) – 38 ˚C; (**d**) – 44 ˚C.

As shown in Figure S14 (a, b, c, d and e) the absolute intensity of the global delayed fluorescence intensity $gI^{dF}$ (after reaching steady state) decreases monotonically.

For the physiologically important temperatures from $T$ = 32 °C up to $T$ = 38 °C, the variation of the ratio of the global luminescence loss, $L_{pO_2}^{dF}$ (at a given oxygen concentration) to the global delayed fluorescence intensity $gI^{dF}$ is less than 5%. (Figure S14 f). This demonstrates the ability for controllably tuning of the $L_{pO_2}^{dF}$ – oxygen calibration curves.

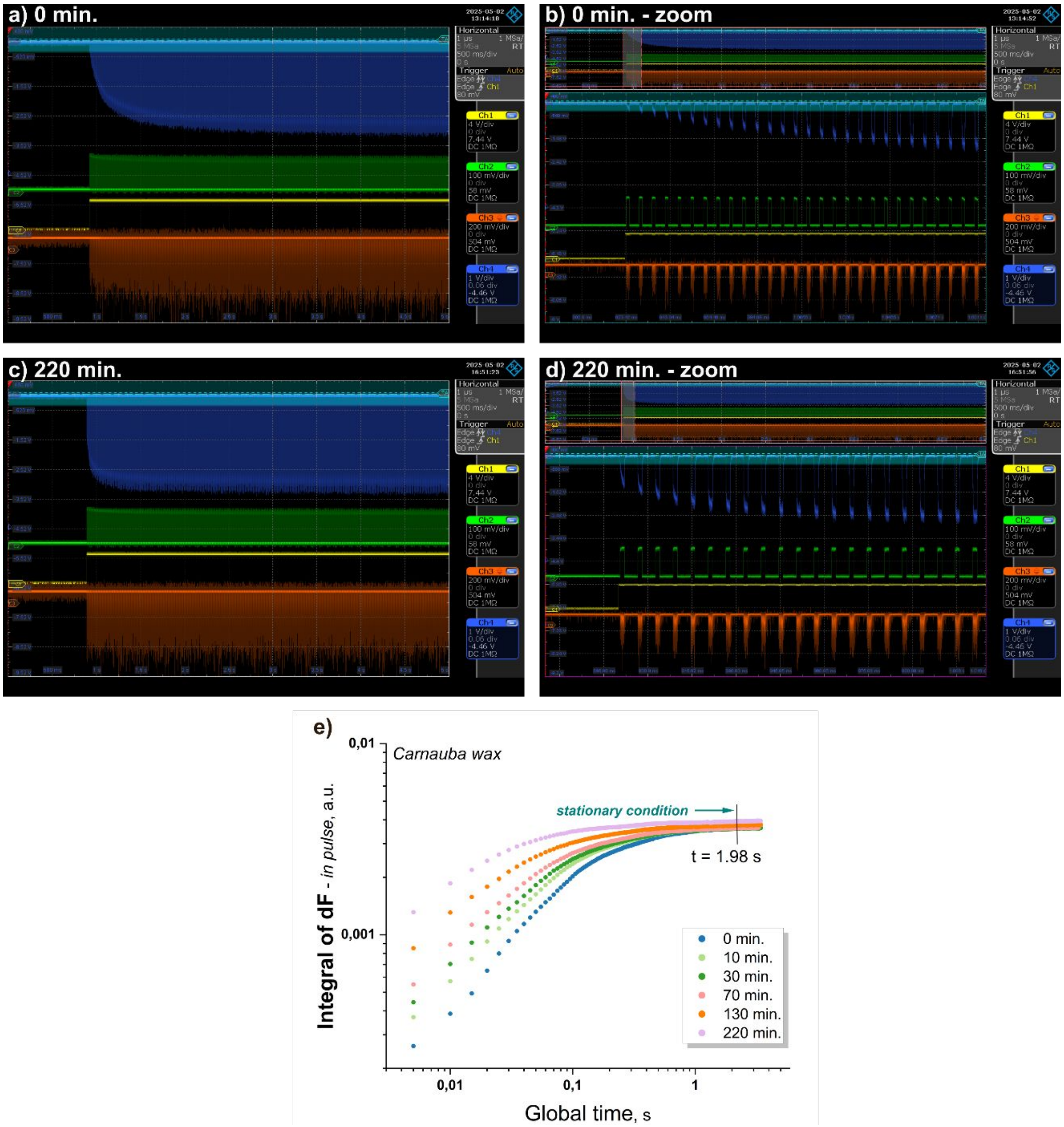


**Figure S15**: (**a**, **c**) Time-resolved intensity of the optical signals of dF and rPh for the TTA-UC molecular system together with the intensity of the excitation laser pulse (the green channel) and measurement start (the yellow channel); (**b**, **d**) – Magnified are the first 25 pulses; (**e**) - Time-dependent $I_{in}^{dF}(n)$ at a constant oxygen partial pressure as a function of the excitation sequence number, during repeated optical excitation of the same sample spot. The time periods shown in the graph.

***NC composition***: The NC composition and preparation technique is identical with the reported in the manuscript, except the 40% wt. beeswax (Sigma Aldrich, CAS Number: 8012-89-3) was replaced with 40% wt. carnauba wax (Sigma Aldrich, CAS Number: 8015-86-9).

***Experimental Conditions***: TTA-UC NC dispersed in water, placed in ambient environment (160 mmHg $O_2$ partial pressure, corresponding to normoxia); sample temperature = 24 ˚C; sample thickness d = $1\times10^{-3}$ m; NC – solid content 5% wt.; peak excitation intensity $I_{exc}$ = 6.6 W×cm$^{-2}$; wavelength $\lambda = 631$ nm; excitation pulse duration $\tau_{pulse}$ = 1.5 ms; excitation pulse period $\tau_{pulse}$ = 6 ms; observation duration $t_{sensing}$ = 4.8 s (800 successive excitation pulses); acquisition time resolution $\tau_{acquisition}$ = 1 µs.

Carnauba wax is a plant-derived wax, with melting point (over 80 °C) much higher than the melting point of beeswax (around 62 °C). TTA-UC nanocapsules containing carnauba wax instead of beeswax exhibit significantly lower oxygen permeability. As shown in Figure S15 e, the oxygen concentration within the NC does not fully recover even after more than 220 min. It is well observable, that even the first excitation pulse (Figure S15 d, excitation sequence number 6) generates significant dF signal, while the first excitation pulse (Figure S15 b, excitation sequence number 1) generates almost no delayed fluorescence.

This behavior is counterproductive when repeated oxygen measurements at relatively short time intervals are required. For instance, beeswax-based NC can be used to perform oxygen measurements at intervals as short as 6 min (Figure S12 f). Conversely, the low oxygen permeability of carnauba wax-based NC minimizes singlet oxygen generation, thereby reducing the consumption of the SSOS materials. This allows multiple sensing runs to be performed (well over 10, compared with beeswax-based NC). Under otherwise identical experimental conditions, the carnauba wax NC reached the steady state significantly faster (t = 1.98 s), providing direct evidence of its lower oxygen concentration.

## *Nonlinear Curve Fit (ExpGro2) (27/04/25 19:31:04)*

### *Notes*

| | |
|---|---|
| Description | Nonlinear Curve Fit |
| User Name | michevam |
| Operation Time | 27/04/25 19:31:04 |
| Iteration Algorithm | Levenberg Marquardt |
| Model | ExpGro2 |
| Number of Parameters | 5 |
| Number of Derived Parameters | 4 |
| Number of Datasets | 1 |
| Equation | y = A1*exp(x/t1) + A2*exp(x/t2) + y0 |
| Report Status | New Analysis Report |
| Special Input Handling | |
| Data Filter | No |

### *Parameters*

| | | Value | Standard Error | t-Value | Prob>\|t\| | Dependency |
|---|---|---|---|---|---|---|
| B | y0 | 5915,8497 | 827898,44119 | 0,00715 | 0,99475 | 1 |
| | A1 | -0,17496 | 0,13651 | -1,28161 | 0,29006 | 0,98821 |
| | t1 | -30,47563 | 35,59154 | -0,85626 | 0,4548 | 0,93747 |
| | A2 | -5915,67206 | 827898,44108 | -0,00715 | 0,99475 | 1 |
| | t2 | -8,57788E6 | 1,20055E9 | -0,00714 | 0,99475 | 1 |
| | k1 | -0,03281 | 0,03832 | | | |
| | k2 | -1,16579E-7 | 1,63162E-5 | | | |
| | Td1 | -21,12409 | 24,67017 | | | |
| | Td2 | -5,94573E6 | 8,32158E8 | | | |

Reduced Chi-sqr = 8.23969846783E-4
COD(R^2) = 0.968607004521
Iterations Performed = 31
Total Iterations in Session = 31
Fit did not converge - mutual dependency exists between parameters.
**You may have overparameterized the fitting function. Fixing one of them may eliminate this p**
Standard Error was scaled with square root of reduced Chi-Sqr.
k1, k2, Td1, Td2 are derived parameter(s).
Some input data points are missing.

### *Statistics*

| | B |
|---|---|
| Number of Points | 8 |
| Degrees of Freedom | 3 |
| Reduced Chi-Sqr | 8,2397E-4 |
| Residual Sum of Squares | 0,00247 |
| R-Square (COD) | 0,96861 |
| Adj. R-Square | 0,92675 |
| Fit Status | Failed(-203) |

Fit Status Code :
-203 : Fit did not converge - mutual dependency exists between parameters.
You may have overparameterized the fitting function. Fixing one of them may eliminate this problem.

### *ANOVA*

| | | DF | Sum of Squares | Mean Square | F Value | Prob>F |
|---|---|---|---|---|---|---|
| B | Regression | 4 | 0,07627 | 0,01907 | 23,14068 | 0,01364 |
| | Residual | 3 | 0,00247 | 8,2397E-4 | | |
| | Uncorrected Total | 8 | 0,16316 | | | |
| | Corrected Total | 7 | 0,07874 | | | |

**B: At the 0.05 level, the fitting function is significantly better than the function y=constant.**

### *Fitted Curves*

B

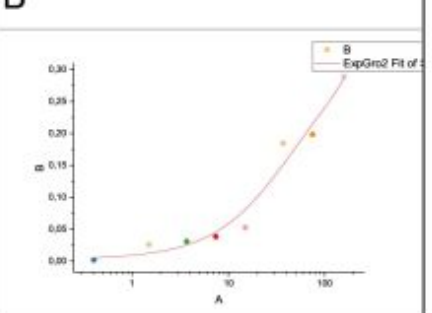

**Figure S16**: Double-exponential fit to the experimental data, shown in Figure 6b.

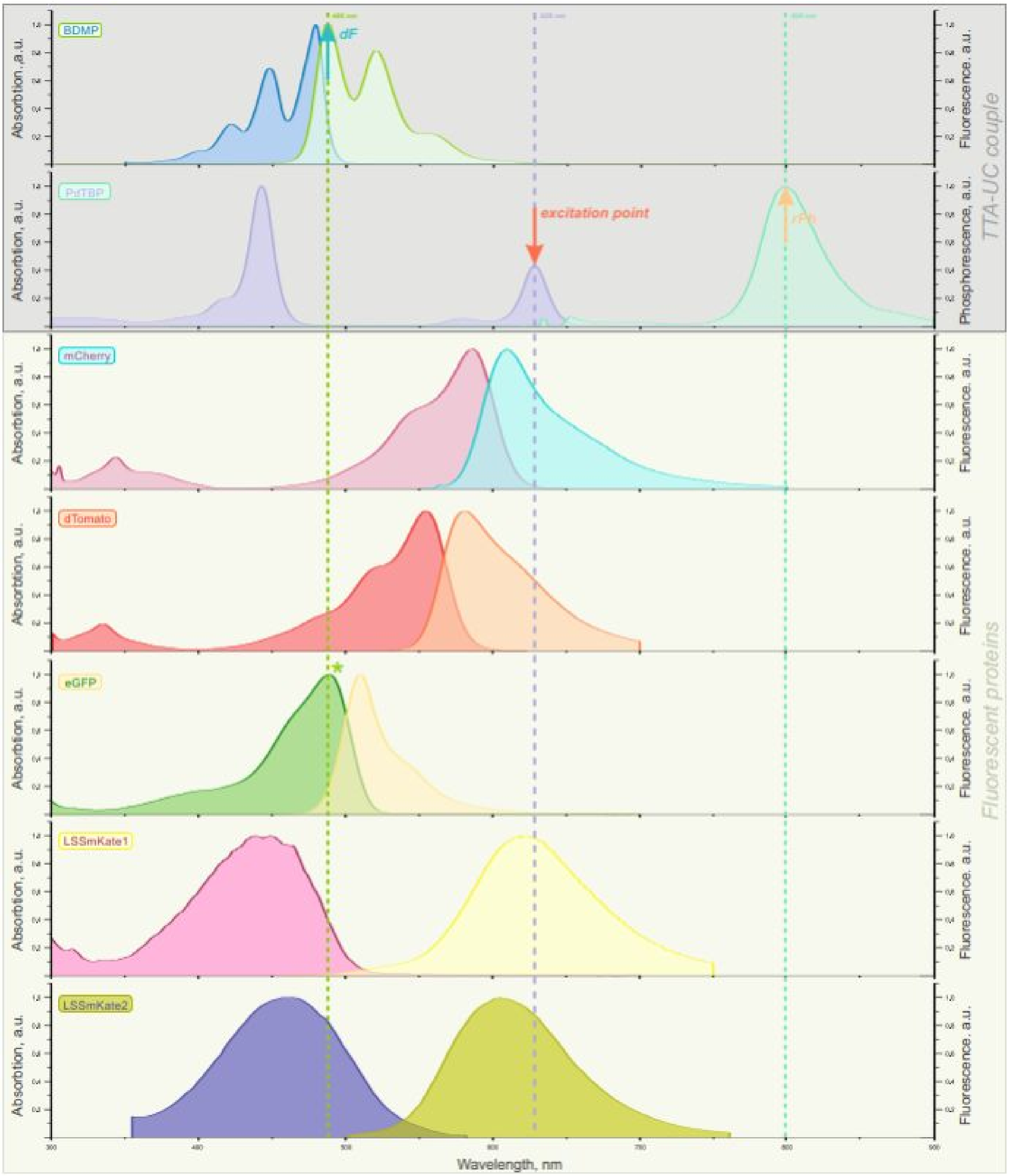


**Figure S17**: Comparison of the excitation and emission spectrum of the process of TTA-UC and group of often used reporter fluorescent proteins - *mCherry*; *dTomato*; *eGFP*; *LSSmKate1*; *LSSmKate2*.

## 3. Materials

**PdTBP** - Meso-tetraphenyl-tetrabenzo[2,3]porphyrin palladium (II) (PdTBP) was synthesized previously by our group [4].

**BDMP** - 3,9(10)-Bis(3,3-dimethylbut-1-ynyl)perylene was synthesized previously from our group.[5]

**Beeswax yellow** - was purchased from Sigma Aldrich Chemie GmbH, Germany.

**Dialysis membrane** Membra-Cel tubes - were purchased from Carl ROTH & Co. KG, Germany.

**RBO** (Rice bran oil) - was purchased from TEA Prodotti Naturali di Manzotti P., Italy.

**Squalene** - was supplied by Thermo Fisher Scientific, Germany.

**Carnauba wax** - was purchased from Sigma-Aldrich Chemie GmbH, Germany.

**Silk fibroin** - 5% solution from domesticated Bombyx mori silkworm (average Mw = 100 kDa) - purchased from Sigma-Aldrich Chemie GmbH Germany.

**OTAC** (n-Octadecyl Trimethyl Ammonium Chloride) - was supplied by Thermo Fisher Scientific, Germany.

**DMEM** (Dulbecco's Modified Eagle Medium, high Glucose, with sodium bicarbonate, L-glutamine and sodium pyruvate, liquid, sterile-filtered, suitable for cell culture) - was purchased from Sigma-Aldrich Chemie GmbH, Germany.

**PBS** (Dulbecco's Phosphate Buffered Saline, Liquid (10X), without $MgCl_2$ and $CaCl_2$, sterile-filtered, suitable for cell culture) - was purchased from Sigma-Aldrich Chemie GmbH, Germany.

**FBS** (Fetal Bovine Serum), Heat Inactivated, non-USA origin, sterile-filtered, suitable for cell culture - was purchased from Sigma-Aldrich Chemie GmbH, Germany.

## References

(1) Filatov, M.A.; Baluschev, S.; Landfester, K. Protection of densely populated excited triplet state ensembles against deactivation by molecular oxygen. *Chem. Soc. Rev*. **2016**, 45, 4668–4689.

(2) Monguzzi, A.; Tubino, R.; Meinardi, F. Upconversion-induced delayed fluorescence in multicomponent organic systems: Role of Dexter energy transfer. *Phys. Rev. B* **2008**, 78, 155122

(3) Roussakis, E.; Li, Z.; Nichols, A. J.; Evans, C. L. Oxygen-Sensing Methods in Biomedicine from the Macroscale to the Microscale. *Angew. Chem. Int. Ed.* **2015**, 54, 8340–8362.

(4) Micheva, M.; Baluschev, S.; Landfester, K. Temperature Sensing in Agarose/Silk Fibroin Translucent Hydrogels: Preparation of an Environment for Long-Term Observation", *Nanomaterials* **2025**, 15, 123.

(5) Wohnhaas, C.; Mailänder, V.; Dröge, M.; Filatov, M.A.; Busko, D.; Avlasevich, Y.; S. Balouchev, S.; Miteva, T.; Landfester, K.; Turshatov, A. Triplet-Triplet Annihilation Upconversion Based Nanocapsules for Bioimaging Under Excitation by Red and Deep-Red Light. *Macromol. Biosci.* **2013**, 13, 1422-1430.
